# Medical Management of Glaucoma in the 21st Century from a Canadian Perspective

**DOI:** 10.1155/2016/6509809

**Published:** 2016-11-08

**Authors:** Paul Harasymowycz, Catherine Birt, Patrick Gooi, Lisa Heckler, Cindy Hutnik, Delan Jinapriya, Lesya Shuba, David Yan, Radmila Day

**Affiliations:** ^1^Université de Montréal, Montreal, QC, Canada; ^2^University of Toronto, Toronto, ON, Canada; ^3^University of Calgary, Calgary, AB, Canada; ^4^Western University, London, ON, Canada; ^5^Queen's University, Kingston, ON, Canada; ^6^Dalhousie University, Halifax, NS, Canada; ^7^SNELL Medical Communication Inc., Montreal, QC, Canada

## Abstract

Glaucoma is a medical term describing a group of progressive optic neuropathies characterized by degeneration of retinal ganglion cells and retinal nerve fibre layer and resulting in changes in the optic nerve head. Glaucoma is a leading cause of irreversible vision loss worldwide. With the aging population it is expected that the prevalence of glaucoma will continue to increase. Despite recent advances in imaging and visual field testing techniques that allow establishment of earlier diagnosis and treatment initiation, significant numbers of glaucoma patients are undiagnosed and present late in the course of their disease. This can lead to irreversible vision loss, reduced quality of life, and a higher socioeconomic burden. Selection of therapeutic approaches for glaucoma should be based on careful ocular examination, patient medical history, presence of comorbidities, and awareness of concomitant systemic therapies. Therapy should also be individualized to patients' needs and preferences. Recent developments in this therapeutic field require revisiting treatment algorithms and integration of traditional and novel approaches in order to ensure optimal visual outcomes. This article provides an overview of recent developments and practice trends in the medical management of glaucoma in Canada. A discussion of the surgical management is beyond the scope of this paper.

## 1. Introduction

In 2009, the Canadian Ophthalmological Society (COS) published the first Canadian evidence-based clinical practice guidelines for the management of adult glaucoma [1]. These guidelines covered various aspects of the disease, including diagnosis, classification, diagnostic tests, management, and follow-up recommendations.

A host of developments in the pharmacological management of glaucoma over the past five years prompted a group of Canadian experts in this therapeutic field to form a scientific panel and review recent evidence. An English-language literature search using PubMed and the Cochrane Library was performed between January 2009 and September 2015 on the topics of open-angle glaucoma and angle closure glaucoma. Meta-analyses, systematic reviews, and Canadian studies were preferred sources. Selected references were reviewed by the scientific panel to ensure their relevance and acceptable methodological quality. During a meeting in Montreal on October 3, 2015, the panel members discussed evidence in the context of Canadian daily practice and relevant changes since the publication of the 2009 COS practice guidelines for the management of glaucoma.

The objective of this article is to provide ophthalmologists with an update regarding advances in the field of glaucoma. It focuses on the aspects that have the potential to influence the use of novel imaging techniques as well as current trends in pharmacological approaches. Surgical management is beyond the scope of this paper and only a brief overview is provided to indicate its potential place in the treatment algorithm.

## 2. Classification and Subtypes

Glaucoma encompasses a variety of conditions with the common feature of an acquired, degenerative optic neuropathy [1, 3]. Glaucoma-related optic neuropathy is characterized by a specific pattern of abnormalities of the optic nerve complex (optic nerve head [ONH], retinal nerve fibre layer [RNFL], and peripapillary region) and corresponding damage to the visual field (VF). Recent evidence indicates that neurodegenerative changes also occur further along the visual pathway [4].

Although glaucoma is frequently associated with elevated intraocular pressure (IOP), an increase in IOP is unnecessary to diagnose this condition [1, 3]. In addition, advances in optic nerve imaging techniques have enabled clinicians to detect structural changes (i.e., loss of RNFL and macular ganglion cells) in patients with borderline elevated IOPs and/or inconclusive observations of disc anatomy [5, 6]. The term preperimetric glaucoma refers to the presence of neural damage in the absence of functional loss sufficient for detection by standard VF techniques. Recently, there has been a great deal of interest and debate regarding the management of patients with preperimetric glaucoma. Further to the general agreement that changes in the optic nerve are permanent, progressive, and associated with reduced quality of life [7], we suggest that frequency of surveillance and diagnostic monitoring of these patients should be increased. One of the limitations in guiding therapeutic decisions for preperimetric glaucoma is that there are no randomized controlled trials in this patient population. Thus, careful surveillance of patients for progression is important. Structural and functional observations from at least five exams are needed to calculate the rate of progression. Frequent reexamination is important to assess the development of new risk factors, such as increased IOP or optic disc hemorrhage that might alter the threshold for initiating therapy.

**Figure 1:**
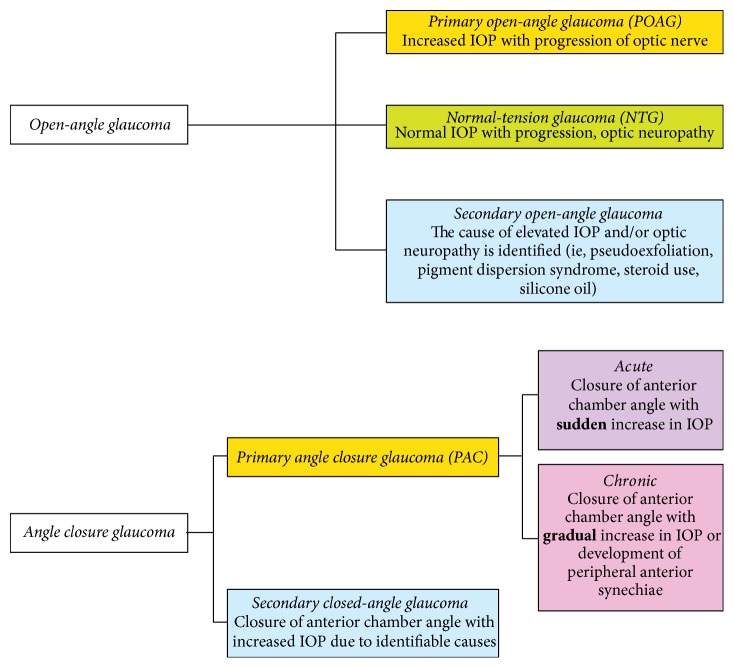
Glaucoma classification and subtypes.

**Figure 2:**
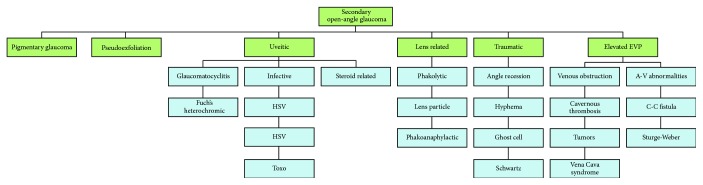
Secondary open-angle glaucoma classification chart.

Glaucoma is classified according to its underlying anatomy and pathophysiology, with open-angle and angle closure representing the two major subtypes, Figure 1 [1, 3]. Both open-angle and closed-angle glaucoma can occur with no identifiable cause, resulting in idiopathic or primary glaucoma. Secondary glaucoma refers to any form of glaucoma in which there is an identifiable cause of increased IOP, resulting in optic nerve damage, Figure 2. Pseudoexfoliative glaucoma is the most common type of secondary glaucoma. Two single nucleotide polymorphisms in the lysyl oxidase-like 1 (LOXL1) gene (rs1048661 and rs3825942) have recently been identified as strong genetic risk factors for pseudoexfoliative glaucoma [8].

Primary open-angle glaucoma (POAG) can be classified based on the age of onset as primary congenital glaucoma (onset up to three years of age), juvenile open-angle glaucoma (JOAG/onset at 3–35 years), and adult-onset POAG (onset after the age of 35 years) [9, 10]. Adult-onset POAG is the most common form and it is a chronic, insidious disease with serious reductions in vision occurring only in the advanced stages. POAG is often but not always associated with elevated IOP due to aqueous humour outflow dysfunction. It has been demonstrated that lowering IOP reduces the rate of glaucomatous damage and consequently loss of VF [1, 3]. In some cases, however, above-average IOP may never lead to neurodegenerative damage, while in others an inherently high predisposition of the optic nerve to damage can lead to optic neuropathy without elevations in IOP (e.g., normal-tension glaucoma [NTG]) [11]. Thus, although all current therapeutic approaches are targeted at lowering IOP as a modifiable risk factor, IOP is not included in the definition of glaucoma; rather it is important for the classification and understanding of the disease.

Normal-tension glaucoma is a form of POAG characterized by glaucomatous optic neuropathy in patients with IOP measurements consistently lower than 21 mm Hg [12–14]. As this subgroup of patients can be challenging to diagnose and treat, some experts consider NTG as a separate entity [14]. Others consider POAG and NTG to exist as a continuum. In general, NTG patients have a higher propensity for optic nerve damage at relatively low IOPs compared to patients with POAG. The amount of VF loss in NTG tends to be greater than one would expect on the basis of optic nerve appearance alone. On ophthalmoscopy, patients with NTG tend to have more localized defects of the retinal nerve fibre layer and an increased tendency for optic disc hemorrhages.

The pathogenesis of NTG remains unclear and it is believed that the interaction of a variety of systemic factors may be involved in the onset and progression of this disease [15]. Recent findings suggest that upregulation of endothelin-1 (ET-1) may be involved in the pathogenesis of NTG and that vascular dysregulation and other IOP-independent mechanisms seem to contribute [16, 17]. Some studies have also postulated a relationship between autoimmune dysfunction and NTG [18, 19], as well as the possible role of intracranial and cerebrospinal fluid pressure [20]. Other observations suggestive of vascular and perfusion abnormalities include the increased prevalence of systemic conditions such as obstructive sleep apnea, migraines, nocturnal hypotension, and Raynaud phenomenon [21, 22].

Primary angle closure glaucoma (PACG) occurs when access to the trabecular meshwork (TM) is physically obstructed, typically by the iris, and the drainage angle is closed [23]. Although a less common form of glaucoma, PACG is a major cause of blindness worldwide due to the severity of the disease, contributing to 50% of the world's blindness from glaucoma [24]. Three main mechanisms hypothesized to be responsible for PACG are pupillary block, anterior iris rotation, and plateau iris [24, 25]. In the former, contact between the iris and the lens increases resistance to the flow of aqueous into the anterior chamber. When the pressure in the posterior chamber exceeds that in the anterior chamber, the iris moves forward and contacts the TM creating aqueous blockage at two levels: the pupillary margin and the TM. Pupillary block is involved in the vast majority of cases of angle closure. In plateau iris configuration, the ciliary body is anteriorly positioned resulting in the anterior displacement of the peripheral iris into the angle [26]. Plateau iris syndrome is characterized by either persistent angle closure or angle closure and elevated IOP in the presence of a patent iridotomy, thus excluding a primary pupil block mechanism. However, the definition of plateau iris and the therapeutic approaches for these patients requires revision in light of our understanding of the significance the lens plays in angle closure [27]. While ultrasound biomicroscopy is the best technique for examining the anatomy of the ciliary body and posterior iris [28], plateau is diagnosed using gonioscopy.

**Figure 3:**
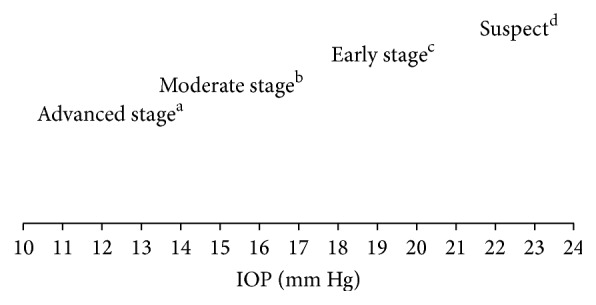
Suggested range for initial target IOP for each eye (a) with ≥ 30% reduction from baseline; (b) with 30% to 35% reduction from baseline; (c) with ≥ 25% reduction from baseline; (d) with ≥ 20% reduction from baseline. In patients with severe optic nerve damage, those with rapidly progressing disease or with other risk factors (i.e., family history, advanced age, pseudoexfoliation, pigment dispersion syndrome, uveitis, steroid use, or disc hemorrhage), selecting target IOP lower than 25% of the pretreatment IOP is justified. Other factors such as the rapidity of progression and the severity of disease in the other eye should be taken into consideration. Conversely, choosing a less aggressive IOP range may be reasonable if the risks of aggressive treatment outweigh the benefits (i.e., comorbidities and older age).

Staging of chronic glaucoma according to its severity is provided in Table 1. This is of particular importance as classifying glaucoma patients according to disease severity and/or rate of progression can help in guiding therapeutic approaches. For example, patients with evidence of rapidly progressing and/or moderate to severe disease should be treated more aggressively from the onset of treatment with IOP targets in the lower end of proposed target ranges, Figures 3 and 5.

As with all glaucoma, open-angle glaucoma can be classified as glaucoma suspect, early, moderate, and advanced depending on the stage of the disease. Recent evidence indicates that retinal ganglion cell (RGC) loss should also be taken into consideration when classifying and staging glaucoma as eyes with the earliest detectable VF loss may already show substantial loss of RGCs [2].

Closed angle can be classified as primary angle closure (PAC), PAC suspect (PACS), and PACG (Table 1(b)). As shown in Figure 1, angle closure can be either acute or chronic. In case of acute PAC, sudden obstruction can lead to rapid rises in IOP and profound acute visual loss and discomfort, requiring urgent evaluation and treatment.

Mixed-mechanism glaucoma has a multifactorial pathophysiology with a number of possible influences. Combined mechanism glaucoma includes characteristics of open and closed-angle glaucoma where, for example, angles that were initially closed open as a result of treatment; however, underlying TM dysfunction results in ongoing elevated IOP. The COS guidelines recommend gonioscopy to determine if the angle is closed, open, or abnormal [1]. Classification of glaucoma on the basis of the appearance of the angle on gonioscopy can help select the appropriate management strategy. Novel anterior-segment imaging techniques can be useful in identifying mechanisms of angle closure and to detect glaucoma damage. Thus, these new techniques are increasingly used to support gonioscopy.

*Takeaway Point. *In addition to traditionally well-defined POAG and PACG, some experts now recognize preperimetric and NTG as two distinct entities. We suggest that newly diagnosed patients undergo frequent initial testing to establish the rate of glaucomatous progression. Patients with unstable pressure, rapid progression, and pseudoexfoliative glaucoma may require closer follow-up and monitoring to ensure earlier intervention in order to prevent permanent optic nerve damage.

Determining the stage of the disease and/or the rate of progression is important from a management standpoint as it helps guide therapeutic decisions and determine which patients require more aggressive treatment.

NTG patients may also require more frequent testing not necessarily because of the burden of their disease but to establish appropriate IOP goals and treatment strategies.

## 3. Interpretation of Recent Epidemiology Trends

It is estimated that 64.3 million people worldwide have glaucoma, of which three-quarters are open-angle [29]. Glaucoma (both open-angle and angle-closure) is the second leading cause of irreversible blindness worldwide, with approximately 8.4 million people becoming blind from the disease [29]. Although any subtype of glaucoma can be found in any ethnic group, angle closure glaucoma is more prevalent among Inuit populations and people of both Asian and south Asian origin (25% in these populations versus <4% among Caucasians or those of African descent) [24]. Individuals of African descent tend to have the highest prevalence of open-angle glaucoma [30]. It is estimated that the number of people with glaucoma globally will increase to 76.0 million in 2020 and to 111.8 million in 2040 [29]. Although there are no Canadian specific estimates, one can assume that similar trends will occur. At the same time, it is important to review and track data specific to the Canadian population toward guiding decisions regarding glaucoma screening, treatment, and public health related strategies, taking into account demographic shifts due to immigration from Asia, the Middle East, and Africa.

A few epidemiological studies on glaucoma and its subtypes in Canada suggest that more than 400,000 people may be affected. More importantly, 50% of glaucoma cases are undiagnosed and not receiving appropriate treatment [31–35]. A recently published cross sectional epidemiological survey in Toronto reported a 4% prevalence rate of undetected glaucoma [32], which is comparable with a previously reported prevalence rate of self-reported glaucoma in Canadians over 50 years of age [33]. Furthermore, according to the study conducted in Toronto, the prevalence rates of POAG and PAC were 3% and 1%, respectively. Narrow angles were found in 15% of participants and 21% had family histories of glaucoma [34]. Although this small-scale study suggested that glaucoma might be more prevalent than previously assumed, it is important to note that it was conducted in a specific population and should be regarded as a proof of concept that warrants larger-scale investigations. Furthermore, the impact of immigration from other parts of the world on glaucoma prevalence needs to be taken into consideration.

In Canada, optometrists are responsible for the majority of referrals for glaucoma [34]. However, the proportion of patients with milder disease referred by optometrists is higher than references for advanced disease (83%, 86%, 80%, and 71% of the optometrist referrals for ocular hypertension [OH] and mild, moderate, and advanced POAG, resp.) [34]. Advanced age [34] and lower socioeconomic status [35] are associated with late presentation. However, further studies to understand other risk factors for late presentation of glaucoma are required as late diagnosis is a risk factor for blindness and thus a significant concern for both the individual and society [36]. In addition, the cost of treating glaucoma rises with increasing disease severity [37]. Thus, although evidence does not support routine screening for glaucoma, except for high-risk populations [38–40], a public awareness campaign that describes risk factors and emphasizes the importance of routine eye examinations should be encouraged. High-risk populations, especially those in remote locations, can be screened and managed via telemedicine (teleglaucoma) [40].

According to a meta-analysis that included 34 studies with over 86,000 participants (range 175 to 6,142) increased cup-to-disc ratio (seven studies; positive likelihood ratio of 14, 95% confidence interval [CI] 5.3 to 39), ratio asymmetry (4 studies; positive likelihood ratio of 7.3, 95% CI 3.3 to 16), disc hemorrhages (5 studies; positive likelihood ratio of 12, 95% CI 2.9 to 48), and IOP (29 studies; positive likelihood ratio of 12, 95% CI 2.9 to 48) were associated with an increased risk of POAG, but their absence did not rule it out [41]. Other risk factors include family history, older age (>60 years), thyroid problems [42], and thinner central corneas. Risk factors for angle closure glaucoma include hyperopia, older age, female gender, Asian, Latino, or Inuit ancestry, and shallow peripheral anterior chamber, which are detailed as follows.

(i) Risk factors for POAG [1, 30, 42]

Race:
  - African people have a prevalence up to 5 times higher than other ethnic groups
  - Hispanic people have a more pronounced increase with age
Age:
  There is an exponential increase with increased age
Refractive error:
  There is an increased risk with high refractive error, both myopia and hypermetropia
Central corneal thickness:
  There is an increased risk with thin central corneal thickness
Optic disc diameter:
  There is an increased risk with a large optic disc diameter
Intraocular pressure:
  There is an increased risk for onset and progression with elevated IOP and a decreased risk for progression with lowering of IOP
Blood pressure:
  There is less risk in young persons with hypertension and increased risk in older persons with hypertension
Cardiovascular disease:
  Cardiovascular disease and glaucoma are probably closely related
Hypothyroidism:
  Thyroid disorders may increase the risk of glaucoma
Physical activity:
  There is an increased risk for low ocular perfusion pressure with low physical activity
Family history

(ii) Risk factors for development of PAC [1]

- Axial hyperopia
- Family history of angle closure
- Advancing age
- Female gender
- East Asian ethnicity
- Inuit ethnicity
- Latino ethnicity
- Shallow peripheral anterior chamber
- Short axial length eyes

The COS recommends that patients exhibiting these factors undergo careful gonioscopy to assess the degree of risk. Follow-up examination of these patients is also important as the risk of developing POAG or chronic angle closure glaucoma (CACG) may increase over time.

Glaucoma also has a significant impact on patients' quality of life from their ability to walk and drive to their ability to read [43, 44]. The psychological burden increases with decreasing vision, along with a growing fear of blindness, social withdrawal, and depression [45]. Measurable loss in quality of life and functionality is observed even in the early stages of the disease and the impact increases as VF loss progresses [46].

Glaucoma also contributes to significant direct and indirect healthcare costs [47–49]. Direct medical costs include ocular hypotensive drugs, physician, and hospital visits, as well as glaucoma-related procedures. Indirect costs reflect lost productivity, such as days missed from work, and include loss of productivity of caregivers. Individuals with late-stage disease incur significant additional indirect costs and constitute a substantial burden on healthcare resources.

*Takeaway Point.* Glaucoma is a common disease affecting approximately 400,000 Canadians and is the leading cause of irreversible blindness, likely responsible for blindness in 54,000 Canadians.

Recent data indicate that a significant number of individuals with glaucoma in Canada remain undiagnosed and present with advanced disease. This highlights the need for public awareness campaigns to provide education on risk factors as well as additional studies to identify causes of late presentation.

## 4. Traditional and Novel Glaucoma Assessment Approaches

Traditionally, the diagnosis of glaucoma has been established by clinical evaluation, including history, slit-lamp biomicroscopy, measurement of IOP, assessment of the anterior chamber angle and optic nerve, and functional VF testing [1, 3].

During the initial evaluation, a complete ocular, family, and systemic history should be obtained. Systemic history should focus on risk factors, particularly in individuals suspected of having NTG, including migraines, history of blood loss, systemic hypertension, sleep apnea, steroid use, and ocular trauma. Elements that might have an impact on future therapy, including systemic hypertension, respiratory, and cardiac disorders, should be noted, particularly the use of systemic *β*-blockers.

The ophthalmic evaluation should include measurement of best-corrected visual acuity (BCVA) and documentation of any refractive error. The pupils should be examined for reactivity and an afferent pupillary defect. A slit-lamp examination of the anterior segment can provide evidence of narrow angles and secondary causes of glaucoma, such as pigment dispersion or pseudoexfoliation syndrome, plus previous angle recession. Findings may include evidence of previous surgery that the patient has forgotten, thus adding a possible game-changing factor to a treatment plan.

Measurement of IOP before dilation is an essential part of the initial examination and must be performed with assurance that breath-holding is prevented. IOP is determined by the balance of aqueous humour production and drainage. Circadian variations in aqueous flow can result in IOP fluctuations of 2–5 mm Hg under normal circumstances [50] and much wider fluctuations for patients with glaucoma [51, 52]. IOP readings should be repeated to provide an accurate picture of the diurnal range [1]. An important role still exists for diurnal IOP curves, in particular with normal pressure glaucoma suspects [53, 54]. Fluctuation in IOP can be detected by taking several pressure readings at different times throughout the course of one day or on different days [55, 56]. A recent study that used modified diurnal tension curves (mDTC; IOP measurements obtained at 8 am, 11 am, 2 pm, and 4 pm on two consecutive days) demonstrated good reproducibility for mean and peak IOP but only fair reproducibility of IOP fluctuation [57]. Thus, serial measurement of IOP in a 24-hour period is still needed to best assess fluctuations in IOP toward optimal glaucoma management [58].

Several devices to measure IOP are available, Table 2 [3], and may be useful in select circumstances. The COS, however, recommends Goldmann applanation tonometry whenever possible, as it is the most reproducible [1]. It is important that clinicians document maximum IOP (*T*_max_) and baseline values as these can serve as a benchmark and reference for future therapeutic goals.

Distinguishing between open-angle and angle closure glaucoma hinges on careful assessment of the anterior chamber angle via gonioscopy. Indentation gonioscopy represents the gold standard of angle evaluation to distinguish appositional from synechial angle closure. A recent Canadian study indicated that a significant number of patients who are referred for cataract surgery present with undetected narrow angles or angle closure, implying that gonioscopy may not be adequately performed in this group of patients and potentially in the general population as well [59]. To that end, imaging modalities such as anterior-segment optical coherence tomography (OCT) and ultrasound biomicroscopy (UBM) can be used to support findings and/or help to distinguish between mechanisms of angle closure. In addition, OCT may more reliably predict PACS patients with progressing disease [60].

Clinically evident characteristics of optic nerve damage and associated VF deficits clearly establish the diagnosis of glaucoma; however, in the early to moderate stages, optic nerve damage can occur without VF loss. Therefore, it is important to document the appearance of the optic nerve. Ophthalmoscopy remains an important aspect of the examination, particularly to identify subtle changes like disc hemorrhages, but stereoscopic disc photographs and computerized images of the nerve are different methods for more objective documentation and analysis of optic nerve morphology [61].

Two commonly used computer-based imaging devices for glaucoma include confocal scanning laser ophthalmoscopy (CSLO) and OCT. These devices provide useful, quantitative information for the clinician when correlated with other relevant clinical parameters and can also be used to monitor progressive changes over time.

The CSLO produces high-contrast retinal images by raster scanning a laser spot and detecting backscattered light through a confocal pinhole [62]. Heidelberg Retina Tomograph (HRT) is commonly used in clinical practice [63]. It is designed to scan the retinal surface with a diode laser, which has a wavelength of 670 nm and can detect changes in the anatomy of the optic disc before VF defects appear. These computerized monitoring and analysis instruments assist clinicians in the detection of changes of the optic disc which are clinically relevant.

Spectral-domain (SD) OCT diagnostic studies have demonstrated that evidence of thinning of the RNFL and ONH structural changes allow for discrimination between glaucomatous and healthy eyes [64]. Evidence to date also suggests high correlations between loss of the ganglion cell complex (GCC) and RNFL and defects on standard automated perimetry (SAP) [65]. A longitudinal SD-OCT study that followed patients with glaucomatous and healthy eyes for three years reported a significantly greater rate of RNFL loss in patients with glaucomatous optic disc progression compared with nonprogressors [66]. As RNFL loss may reach a plateau in advanced disease [67], macular parameters may be more useful for detecting progression in this challenging subset of patients [68].

One disadvantage of these new techniques is that they are based on comparisons to normative databases and therefore may not represent all patients. Age-related loss of neuroretinal parameters also needs to be taken into account [69]. In addition, it can be difficult to distinguish normal findings attributed to myopia and partial colobomas from those of the glaucoma. The diagnostic performance of these instruments and their ability to detect progression are expected to continue to improve as the technology evolves.

Automated static threshold perimetry is the technique of choice for evaluating VFs [70]. VF testing based on the frequency doubling technology (FDT) [71] may detect defects or progression of defects earlier than conventional white-on-white perimetry in some patients [72] and thus can be useful in screening for glaucoma. SAP techniques focus on the central 24° or 30° of vision but do not take into account the peripheral fields, which may have functional relevance. Central 10-2 fields, important in advanced disease, could also be considered in earlier stages of the disease, as evidence has shown that central defects can be missed with the 24-2 test [73]. More frequent VF testing earlier in the follow-up period can determine rates of progression and identify rapid progressors [74]. It is essential to retest patients to confirm defects and to assess the rate of progression by comparing changes in VF over time. Both event-based (detects progression when a follow-up measurement exceeds a preestablished threshold for change from baseline) and trend-based (detects progression by evaluating the slope of measured parameter over time) approaches have advantages and disadvantages [1]. However, clinical judgement and integration of all findings should always supersede computer-based progression.

Kinetic Goldman perimetry may also be of use in patients in whom an automated VF cannot be reliably completed or if more peripheral VF damage is suspected (e.g., temporal wedge).

Finally, the use of neuroimaging, including computed tomography (CT) scan and magnetic resonance imaging (MRI) is indicated in specific cases to establish a diagnosis and to rule out compressive lesions that can mimic glaucoma. Recent evidence has also suggested roles for ancillary testing, including 24-hour blood pressure monitoring, diastolic ocular perfusion pressure [75], and translaminar pressure difference [76].

*Takeaway Points*

- Evaluation of patients with glaucoma and those in whom glaucoma is suspected should include relevant ocular and systemic medical history.
- IOP readings should be done by Goldmann applanation tonometry whenever possible and should be repeated to obtain an accurate estimate of the diurnal range.
- RNFL analyses are now a part of diagnosis and follow-up. New instruments of computerized monitoring and analysis can assist clinicians in the detection of changes of the optic disc that are clinically relevant.
  - CSLO and SD-OCT produce high-contrast retinal images and can detect changes in the anatomy of the optic disc before VF defects appear.
  - Newer imaging modalities such as anterior-segment OCT and UBM can be used to distinguish between mechanisms of angle closure and predict progressive angle closure.
  - SD-OCT technologies continue to advance and are helpful in establishing diagnosis and monitoring for progression.
- Standard automated perimetry using 24-2 or 30-2 techniques remains the test of choice for evaluating functional field loss in glaucoma. Central 10-2 fields, important in advanced disease, could also be considered in earlier stages of the disease to ensure that central defects are not missed.

## 5. Treatment Goals

Glaucoma management is aimed at reducing IOP, the only known modifiable risk factor at this time. In some individuals, however, systemic factors such as uncontrolled systemic hypertension, vasospasm, sleep apnea, and arrhythmias may play a minor or major part in the development of glaucoma. The ultimate goal is to slow or stop structural and functional progression while maintaining or enhancing overall quality of life. Some recent evidence also suggests that VF improvement may be achieved with IOP lowering [77]. The treating ophthalmologists should strive to maintain the IOP in a stable range to prevent further damage of the optic nerve [78]. Achievement of targeted IOP might require aggressive treatment and frequent change of therapy; however, the target IOP range is a dynamic concept and it should be individualized and constantly reevaluated, taking into consideration stage of disease, patient risk factors, life expectancy, and social circumstances. Furthermore, the means by which IOP targets are achieved can also be customized, and consideration of medications, laser, and surgical options may be required based on the patient's individual characteristics and circumstances.

For the selection of the initial target IOP range, the COS suggests that each eye is staged into one of four severity groups: suspect, early, moderate, or advanced glaucoma [1]. The severity groups are based on assessment of the optic nerve and/or VF. However, recent evidence suggests that baseline and longitudinal estimates of RGC counts may be helpful in predicting progression and performed significantly better than conventional approaches for risk stratification of glaucoma patients [79, 80]. The suggested initial target IOP range for each eye is provided in Figure 3, which is based on the COS recommendations [1] and the authors' experience.

Lowering the pretreatment IOP by ≥25% slows down progression of glaucoma in many, but not all, patients [81–83]. In general, treating clinicians should select an initial target pressure at least 25% lower than pretreatment pressure for patients in the early stages of the disease and at least 30% for patients with moderate and advanced disease. An absolute IOP target, based on the stage of the disease, can also be used as a guide, Figure 3. However, over the past few years an emerging trend toward lower IOP goals than those suggested by COS guidelines has emerged. The goal is to treat aggressively from the beginning to prevent further damage and preserve vision. In addition to targeted pressure, it is also recommended to take diurnal pressure fluctuations into consideration when selecting therapeutic approaches. In patients with severe optic nerve damage, those with rapidly progressing disease or with other risk factors (i.e., family history, advanced age, pseudoexfoliation, pigment dispersion syndrome (PDS), uveitis, steroid use, or disc hemorrhage), selecting target IOP lower than 25% of the pretreatment IOP is justified [1, 3, 78]. Other factors such as the rapidity of progression and the severity of disease in the other eye should be taken into consideration. Conversely, choosing a less aggressive IOP range may be reasonable if the risks of aggressive treatment outweigh the benefits (i.e., comorbidities and older age).

As glaucoma patients might continue to progress despite treatment even if their IOP levels are within targeted range, relying on tonometry alone for glaucoma follow-up is insufficient [3]. Determining the rate of VF progression is a new standard in glaucoma care and the general recommendation is to perform six VF examinations in the first two years [74, 84]. However, taking into consideration the lack of resources and the burden on patients (time of work, travelling, etc.) patients could be stratified according to initial field defects and/or risk factors for progression [85]. Those with evidence of more damage and higher risk of disease progression should be monitored more frequently.

*Takeaway Points*

- Taking into consideration ocular characteristics (pseudoexfoliation, PDS, and uveitis) as well as patient-related factors such as risk factors, comorbidities and life expectancy are necessary in determining a patient's target IOP range.
- Every patient is unique, and physicians must customize the means by which they achieve IOP ranges accordingly, taking into consideration the use of any combination of drops, laser, and surgery in order to best achieve the therapeutic IOP goal while minimizing the impact on patient quality of life.
- Target IOPs should be revisited and adjusted frequently. For example, it is appropriate and often necessary to set lower IOP targets in patients with progressive disease. In patients with stable disease or major changes in their overall medical situation it may be appropriate to allow a higher IOP target.
- There is recent evidence that IOP lowering may improve VF defects [77].

## 6. Contemporary Pharmacological Management of Glaucoma

**Figure 4:**
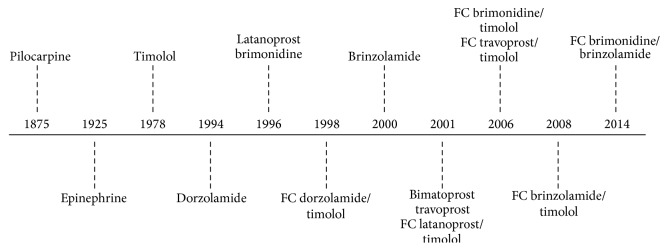
The chronology of the introduction of topical IOP-lowering medications. Systemic carbonic anhydrase inhibitors have been available since 1955.

Unless contraindicated, medical therapy remains the most common initial method of lowering IOP and usually involves topical agents delivered as eye drops [1, 3]. There are several effective classes of topical therapies for glaucoma, including prostaglandin analogues (PGAs), *β*-blockers, *α*-adrenergic agonists and carbonic anhydrase inhibitors (CAIs), and pilocarpine, Figure 4. These topical therapies reduce the production of aqueous humour, enhance its outflow, or have an effect on both. For many years, topical *β*-blockers were the most commonly used first-line medical therapy; however, the introduction of newer agents over the past 20 years has given patients and physicians a wider variety of choices for both initial and adjunctive treatment. However, some of the “older drugs,” such as the parasympathomimetic agent pilocarpine and oral CAIs, still play a significant role in specific types of glaucoma, including plateau iris.

### 6.1. Prostaglandins: First Choice for Glaucoma Treatment

The combination of effectiveness and tolerability has made topical PGAs such as latanoprost, bimatoprost, and travoprost popular first choices for treating glaucoma. PGAs have demonstrated better IOP-lowering ability than *β*-blockers with fewer systemic adverse effects [86–88]. They act by increasing uveoscleral and TM outflow and reduction in IOP starts 2–4 hours after first administration. The therapeutic effect reaches its peak after 8–12 hours. These agents also minimize IOP fluctuations during a 24-hour period, with a maximum effect achieved 3–5 weeks after initiation of therapy.

Several studies have shown that topical administration of travoprost leads to a mean IOP reduction from 25% to 32%, which is sustained throughout the 24-hour cycle [88, 89]. In a prospective, open-label, single-arm study conducted in Italy (*N* = 36 previously untreated POAG patients), travoprost monotherapy at a dose of 0.004% administered once in the evening (8:00 pm) induced uniform 24-hour IOP reduction. This was maintained during the 5-year follow-up of the study (range of 24-hour IOP reduction: 27.8%–28.6%) [89]. Although mean nocturnal IOP reduction with travoprost was somewhat lower than mean daytime IOP reduction, there was no significant difference between nighttime and daytime efficacy [89]. A preservative-free formulation of travoprost 0.004% is available to reduce tolerability-related problems in subjects affected with ocular surface disease [88].

Although prostaglandins have an excellent systemic safety profile, they are associated with several cosmetic ocular adverse effects that might be of particular relevance to younger patients with unilateral disease. These include conjunctival hyperemia, elongation and darkening of eyelashes, and induced iris darkening. Periocular skin pigmentation and fat atrophy can result in a sunken looking appearance, referred to as prostaglandin-associated periorbitopathy that includes deepening of the upper eyelid sulcus, upper lid ptosis, enophthalmos, and loss of the inferior orbital fat pads [90, 91]. To that end, some clinicians offer bilateral treatment to patients despite being indicated in one eye, to provide symmetrical adverse effects.

### 6.2. *β*-Blockers: Conceptions and Misconceptions

With over 30 years of clinical use, topical *β*-blockers, indicated for once (QD) or twice daily (BID) use, have a proven efficacy and known contraindications. As *β*-blockers are systemically absorbed, they were historically contraindicated in patients with cardiac or pulmonary disease [92]; however, some evidence suggests that *β*-blockers may be better tolerated than originally thought in patients with these conditions [93, 94]. Thus, with careful patient monitoring and follow-up, *β*-blockers may still present a valuable option for a majority of these patients. This is of particular importance as almost all fixed-dose combinations contain a *β*-blocker, which traditionally limited the therapeutic choices for a significant number of glaucoma patients. In light of newer non-*β*-blocker combinations (see below), however, clinicians should also consider other potential light contraindications to *β*-blockers such as lack of energy and fatigue.

Studies conducted in the 1980s and 1990s suggested that, in certain patients, treatment with *β*-blockers can lead to a rapid increase in the density of *β*-adrenergic receptors on the cell surface, which subsequently can lead to tachyphylaxis (sometimes within a day of the initial dosage) [95–97]. Although tachyphylaxis did not emerge as a significant concern in long-term studies with the *β*-blocker timolol, in an era when alternative therapies are available, awareness of the possibility of tachyphylaxis with *β*-blockers is important for assessing appropriate dosage and response to therapy [98, 99].

Another question surrounding the use of *β*-blockers relates to their dosing and timing of administration. As it is well established that a circadian variation exists in the rate of aqueous humour production, with the rate approximately 50% lower at night compared to daytime [100, 101], it is intuitive that the morning administration of *β*-blockers may provide more benefit. Due to conflicting results of available trials [102, 103], however, it is left to treating clinicians to tailor the dose and administration according to patient characteristics and needs. To that end, it is important to note that the differences in the mean IOP reductions between QD administration of 0.25% and that of 0.5% timolol were not statistically significant. Thus, a lower concentration of timolol used QD may achieve maximum IOP reduction.

### 6.3. *α*-2 Agonists: Effects on IOP

*α*-2 Agonists decrease IOP by the constriction of the afferent ciliary vasculature, leading to decreased aqueous humour production and also by increasing uveoscleral outflow [104]. A meta-analysis indicated that the *α*-2 agonist brimonidine is effective as an IOP-reducing agent with an ability to reduce baseline IOP by approximately 17% [105]. Available evidence and experience from routine practice suggest that *α*-2 agonists are relatively safe long-term IOP-reducing agents, although ocular allergy may lead to discontinuation in approximately 10%–20% of patients [104].

### 6.4. CAIs: Use in Patients with Sulfa Allergy

Recent evidence also points to potentially wider use of CAIs, especially since the misconceptions about contraindications in patients with proven or putative sulfa drug allergies have been clarified [106–108]. The immune components of the antibiotic sulfonamides are not present in the nonantibiotic sulphonamides, including CAIs. According to Strom et al. [106] an association between previous hypersensitivity following the administration of sulfonamide antibiotics and a subsequent allergic reaction after that of a sulfonamide nonantibiotic is due to a predisposition to allergic reactions rather than to cross-reactivity with sulfonamide-based groups. This indicates that patients with a documented sulfa allergy might still benefit from CAIs with proper monitoring and follow-up. Pharmacists and other healthcare professionals involved in the care of patients with glaucoma should also be made aware of this recent evidence.

### 6.5. Fixed Combination Therapy

As it is currently recommended to use the least amount of medication to achieve the desired IOP reductions, the European Glaucoma Society (EGS), National Institute for Health and Care Excellence (NICE), and COS guidelines recommend monotherapy as first-line treatment of glaucoma [1, 3, 109]. However, many patients require more than one agent to reach the desired target IOP. For example, in the Ocular Hypertension Treatment Study (OHTS), 40% of patients randomized to treatment required ≥2 medications to achieve their target IOP at five years [110].

Although a systematic review and meta-analysis that included 18 trials and assessed fixed- and variable-dose combinations of PGA and timolol demonstrated that the fixed-dose combinations might be less efficacious than variable-dose combinations (mean difference in the reduction in IOP from baseline was 0.69, 95% CI: 0.29 to 1.08), the former was associated with a lower risk of hyperemia (relative risk: 0.70, 95% CI: 0.43 to 1.14) [111]. Fixed-dose combinations have also been found to confer additional benefits (described below) compared to variable-dose combinations.

Clinicians should also strive to utilize the minimum number of medication bottles with the minimum dosing frequency to achieve the IOP target. This therapeutic approach may carry several benefits over the concurrent administration of two distinct medications, including the reduction in ocular exposure to preservatives.

 Benefits of fixed-dose combinations [112, 113] are as follows:

- Avoidance of the potential for washout of the first drug by the second.
- Patient convenience of having only one medication bottle and a reduced number of eye drops to dispense.
- Potentially lower cost as a result of fewer copays.
- Reduction in ocular exposure to preservatives.

Ocular preservatives contained in topical formulations have been implicated in the development of ocular surface disease in patients with glaucoma [114, 115]. Increased preoperative exposure to ophthalmic solutions preserved with benzalkonium chloride (BAK) is also a risk factor for earlier surgical failure regardless of the number of medications used [116]. However, one should also keep in mind the potential negative effect of certain combination therapies that include *β*-blockers and/or *α* agonists on diastolic ocular perfusion pressure (DOPP), especially in NTG patients and in smaller patients or children [117, 118]. Awareness of the potential impact of the topical medications on blood pressure, especially the nocturnal diastolic blood pressure, is important. A study that included 27 previously untreated patients with POAG demonstrated that treatment with the timolol-dorzolamide fixed-dose combination (TDFC) led to lower 24-hour IOP compared to latanoprost (mean ± SD: 15.4 ± 1.9 versus 16.7 ± 1.7 mm Hg; *P* < 0.004) [119]. Mean 24-hour SBP and DBP were significantly reduced with TDFC but not with latanoprost. Both treatments significantly increased 24-hour DOPP—the increase from baseline was 5.9 mm Hg (95% CI: 5.3 to 6.5) with TDFC and 6.5 mm Hg (95% CI: 5.8 to 7.1) with latanoprost—with no difference between the two medications. Thus, it appears that enhancement in 24-hour DOPP by TDFC is due to counteracting the decrease in DBP with a substantial reduction in IOP [119].

It had also been suggested that DTFC may provide better 24-hour efficacy than brimonidine/timolol fixed combination (BTFC) in primary open-angle glaucoma (POAG) [120].

The studies and examples provided above indicate that most fixed combinations include the *β*-blocker timolol with a second component, either a PGA, CAI, or *α* agonist.

The most recent fixed-dose combination entry on the Canadian market is brinzolamide/brimonidine. This first non-*β*-blocker combination provides an additional, cost-effective option that is expected to have a positive impact on patient adherence. Both brinzolamide and brimonidine have been used as part of other fixed combination therapies (with timolol), and patients on both have experienced clinically relevant IOP reductions when using these agents adjunctively with a PGA [121–123]. In two Phase III randomized controlled trials in individuals with glaucoma, brinzolamide/brimonidine eye drops were statistically significantly superior to either constituent drug administered alone as monotherapy in reducing IOP at three months [124, 125]. The combination eye drops were noninferior to brinzolamide plus brimonidine administered concomitantly [126, 127].

### 6.6. Generic Fixed Combination Drugs

Although it is generally accepted that generic drugs are bioequivalent to that of brand-name drugs, a study of latanoprost showed that the IOP-lowering effect of the brand-name drug was better compared with the corresponding generic drug in POAG and ocular hypertension patients [128]. The difference in IOP lowering could be due to the difference in adjuvants and/or the stability of the active ingredient once the bottle is opened [128]. One study demonstrated up to 40% loss of concentration of generic latanoprost within 30 days at room temperature after the bottle was opened compared to 6% for the brand product [129]. Although generic drugs have the same quantitative compositions in terms of active ingredients, a difference in adjuvants translates to differences in viscosity, surface tension, and pH, which could affect efficacy and safety [130–132]. For example, the pH of generic dorzolamide/timolol is much higher than branded dorzolamide/timolol, despite the fact that the original studies on dorzolamide in the 1990s showed that the lower pH was essential for corneal penetration and efficacy [133]. In addition, it is believed that the differences in symptoms of discomfort could be influenced by the drop volume, which can be attributed to different bottle designs [132].

### 6.7. The Need for Novel Therapies

Although there have been improvements in formulations and fixed combination therapies, no novel class of drugs for the treatment of glaucoma has been approved since latanoprost in 1996. Furthermore, despite recent advances, currently available medical therapy often fails to meet the desired outcomes and ultimately cure the disease. Hence, there is a need for effective alternatives that have a longer duration of action and offer patients simple dosing regimens.

An example of several emerging therapies is the *ρ* kinase inhibitor [134–136]. *ρ* kinase is a serine/threonine kinase that plays a key role in regulating the contractile tone of smooth muscle tissues in a calcium-independent manner, directly targeting aqueous humour trabecular outflow. Animal models have demonstrated that *ρ* kinase inhibitors reduce IOP by enhancing aqueous humour drainage through the TM and may also lower episcleral venous pressure (EVP) [135, 136].

Adenosine receptor agonists are also able to increase conventional aqueous outflow and are being investigated as IOP-lowering agents [137, 138]. These agents may induce cell shrinkage and secretion of metalloproteases in human trabecular meshwork, resulting in remodeling of the extracellular matrix and reduced outflow resistance [139]. Another novel agent targeting the trabecular meshwork is latrunculin B. In patients with ocular hypertension or early POAG (phase I clinical trial) twice daily latrunculin B (0.005%, 0.01%, 0.02%, or 0.05% solution) significantly lowered IOP compared with contralateral, placebo-treated eyes, with few and mild ocular adverse events [140].

Different drug formulations and delivery methods are also being investigated with a goal to reduce inconvenience associated with topical drug delivery. Some of the innovative methods include punctal or tear duct plugs, topical ring inserts, subconjunctival injections and inserts, and intraocular inserts [141].

Several approaches that involve neuroprotective agents and interventions are being explored [142]. Neuroprotection for glaucoma refers to any intervention, independent of IOP reduction, which protects the optic nerve or prevents the death of retinal ganglion cells. Although significant evidence from preclinical studies has suggested a potential role of neuroprotectors (such as brimonidine or memantine) in the prevention of glaucomatous degeneration, clinical research on the neuroprotective effects of oral and topical medical therapy for glaucoma in adults has been inconclusive.

*Takeaway Points*

- The combination of effectiveness and tolerability has made topical PGAs such as latanoprost, bimatoprost, and travoprost popular first choices for treating glaucoma.
- Despite advances in medical therapy, “older drugs” such as the parasympathomimetic agent pilocarpine still play a significant role in specific types of glaucoma including angle closure.
- Recent evidence also shows that CAIs can potentially be used in more patients, especially since the misconceptions regarding contraindications in patients with proven, or putative, sulfa drug allergies have been clarified.
- Recent evidence suggests that with careful monitoring topical *β*-blockers may present a reasonable therapeutic option in patients with cardiorespiratory conditions.
- Fixed combination therapies provide a convenient and cost-effective means of advancing patient therapy, decreasing ocular exposure to preservatives and they have the potential to improve patient compliance.
- Although generic drugs have the same qualitative and quantitative compositions in terms of the active ingredient, a difference in adjuvants can lead to differences in drug stability, viscosity, surface tension, and pH, which could affect efficacy and safety.
- The *ρ* kinase inhibitors, currently in Phase III clinical development, represent a promising new class of therapy.

## 7. Treatment Algorithms

When selecting initial medical therapy for a glaucoma patient it is important to take into consideration his/her medical history, risk factors, likelihood of compliance, and known allergies that might interfere with topical therapy [1]. Furthermore, ocular surface disease and presence of concomitant eye disease can be detrimental in deciding on a specific type of topical therapy as many of the currently available preparations contain BAK [143, 144]. This antimicrobial agent can damage the ocular surface of the eye, producing conjunctival inflammation, tear film instability, and corneal cytotoxicity. Due to the chronic nature of their disease, which requires dosing of topical medications over many years, glaucoma patients tend to have a higher prevalence of ocular surface disease than the general population [145]. A travoprost/timolol fixed-dose combination without benzalkonium chloride has been found to be effective in achieving IOP control while offering protection to individuals who have relative or absolute contraindications to exposure to this compound [146]. Other preservative-free products are available.

As mentioned, there is no specific target for lowering IOP and the target and monitoring intervals vary according to the severity of the disease and risk of progression [1, 3, 78, 109]. For example, a patient with signs of disease progression and IOP greater than the established target range requires a change in treatment plan and monitoring every 1-2 months, whereas a patient with no signs of progression and with an IOP within the set target range requires no change in plan and monitoring can be extended to every 6–12 months [109].

**Figure 5:**
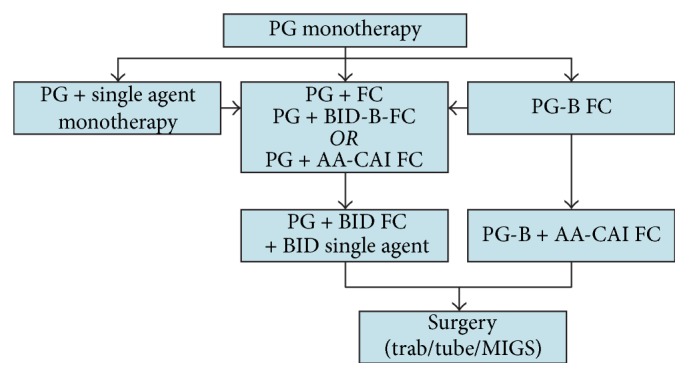
Glaucoma pharmaceutical management algorithm (SLT, MIGS, or other surgeries may be performed at any step of the algorithm). FC, fixed combination; BID single agent, dorzolamide, brinzolamide, and brimonidine; PG, prostaglandin analogue (latanoprost, travoprost, and bimatoprost); PG-*β*B FC, prostaglandin + timolol (latanoprost + timolol, travoprost + timolol); BID-*β*B FC, BID-dosed combination with timolol (dorzolamide + timolol, brinzolamide + timolol, brimonidine + timolol); AA-CAI F, *α* agonist + CIA (brimonidine + brinzolamide); SLT, selective laser trabeculoplasty; MIGS, microinvasive glaucoma surgery. Patient and disease characteristics should be considered at all stages of algorithm and the therapy should be individualized according to patient needs (i.e., ability to tolerate any component of the therapy). In addition, at all stages of the treatment algorithm it is imperative to monitor for adverse effects as well as disease progression (RNFL/VF/disc).

Currently, monotherapy with a topical PGA is considered a first-line medical therapy, Figure 5, withstanding other considerations such as cost, adverse effects, intolerance, or patient refusal. Among the four different classes of topical therapies most commonly used in Canada, PGAs have shown the greatest efficacy while having the lowest dosing frequency (QD). A patient with severe disease and high IOP might benefit from a fixed-dose combination at initiation of therapy.

If the initial therapy is ineffective and the target pressure range (usually 25%–30% for first-line therapy) is not reached or the drug is not tolerated, a patient should be switched to another monotherapy or combination therapy, depending on other risk factors, VF defects, or ONH damage. However, patient compliance should be assessed prior to switching or adding a new therapy. In addition, at all stages of the treatment algorithm it is imperative to monitor for adverse effects as well as disease progression in the VF and/or optic disc as well as RNFL. In case of disease progression, target IOP level and therapeutic options should be adjusted to prevent further progression.

A proposed treatment algorithm for stepwise addition of medical therapy is shown in Figure 5. Although it is generally preferable to introduce one agent at a time to properly assess the efficacy of each drug, it is accepted that there are scenarios when it may be more advisable to start with a fixed combination. Consider a patient presenting with an extremely high baseline IOP and significant nerve damage who is not likely to reach target IOP on a single agent. Once a patient has been treated with a topical PGA but is in need of additional IOP lowering, there are few options: (1) add another single agent, (2) add a combination agent, or (3) switch to a PGA + *β*-blocker fixed combination. The options with combinations are generally preferable with regard to compliance and convenience to the patient. In the less common instance where a patient cannot tolerate a *β*-blocker, it may be necessary to add a BID-dosed single agent in a second bottle without timolol (e.g., dorzolamide, brinzolamide, brimonidine, and pilocarpine). As to which combination to use one might consider that both CAI [147] and *α*-2 agonists [148] have better ability to lower IOP than *β*-blockers. Thus the selection of the second-line agent might depend on reduction of IOP achieved with the first-line PGA.

The concept of maximum tolerated medical therapy (MTMT) in glaucoma can be defined as the achievement of the greatest possible IOP reduction with largest number of medications that the patient can tolerate and is willing to be compliant in administering regularly. Thus, the first step in maximizing medical therapy is to make sure that a patient can adhere to the regimen, as an increase in the number of medications is often associated with decreased compliance. To that end, fixed-dose combinations are particularly useful in that they reduce the number of products and dosing and, as such, cause less interference with the patient's daily activities. Assuming that a patient can tolerate taking all four of the commonly used classes of glaucoma medications in Canada (PGA, *β*-blocker, CAI, and *α* agonist), two different combinations can be employed to achieve MTMT: (1) PG + BID-dosed fixed combination with timolol + BID single agent without timolol or (2) PG-*β*-blocker + *α*-agonist/CAI fixed combinations. The PG-*β*-blocker + *α*-agonist/CAI fixed combination has the advantage of fewer bottles (two versus three) and fewer drops (three versus five) compared to the first MTMT cocktail (PG + BID-dosed fixed combination with timolol + BID single agent without timolol) which was most commonly used prior to the introduction of the *α*-agonist/CAI fixed combination. Pilocarpine and oral CAIs may also be added in order to achieve a true MTMT.

When it becomes necessary to increase therapy beyond a PG + fixed combination, a BID-dosed single agent can be added to achieve MTMT. With the availability of an *α*-agonist/CAI fixed combination, it is also possible to simply add this combination to the PG + *β*-blocker fixed combination to achieve MTMT. This has the advantage of requiring fewer changes in therapy to achieve MTMT, as well as the convenience/compliance advantages of fewer drops/bottles.

At all stages in the treatment algorithm, one should consider laser trabeculoplasty or MIGS (see below) as an alternative to adding medication when additional IOP-lowering is required. It is generally recommended that such alternate modalities be employed earlier in the treatment algorithm before reaching MTMT, as they are not considered substitutes for decisive invasive surgery with either filtering procedure or a tube shunt.

Recent evidence suggests that monocular therapeutic trials might be poor tests of treatment efficacy due to asymmetric spontaneous IOP fluctuations [149–152]. The monocular trial can provide useful clues as to whether a medication is effective but should not be the only information used to guide therapeutic decisions. To ensure valid results, where possible, multiple pressure checks should be performed before and after starting a new therapy.

Introduction of laser trabeculoplasty earlier in the treatment algorithm (i.e., after first-line medical therapy) is another valid therapeutic approach. More than 20 years ago, the Glaucoma Laser Trial found argon laser trabeculoplasty to be as effective as medication for treating newly diagnosed POAG [152]. Since then several studies have looked at the efficacy of selective laser trabeculoplasty (SLT) versus medical therapy. The SLT/MED study found no statistically significant differences in IOP reduction after 9–12 months of follow-up between the SLT and prostaglandin analogue therapy [153]. Micropulse laser trabeculoplasty (MLT), a relatively new laser procedure, can be considered as an alternative to SLT. MLT uses a specific diode laser to deliver laser energy in short microbursts [154]. It aims to provide similar IOP reduction with reduced risk of IOP spikes, making it more attractive for young patients or those with advanced disease who might be at higher risk for IOP spikes.

Over the past decade, traditional glaucoma surgery has been augmented by the advent of innovative techniques and new implants. These new procedures and devices aim to lower IOP with a higher safety profile than that possible with fistulizating surgery and are collectively referred to as microinvasive glaucoma surgery (MIGS). Currently, MIGS is performed in patients with early to moderate disease (similarly placed in the algorithm as SLT) and preferably in combination with cataract surgery [155]. Thus, MIGS is often used to postpone a more invasive surgical intervention in the early to moderate stages of glaucoma and to improve patient adherence to treatment and overall quality of life. In addition, MIGS can possibly be used in patients with advanced disease and those refractory to previous glaucoma-filtering surgeries [156], although this still requires further study.

Many of the current MIGS devices are Schlemm canal devices (SCD) [157], intended to enhance conventional outflow, and assume an intact and functioning posttrabecular outflow system. We propose a newer classification scheme to glaucoma that places a greater emphasis on the pathophysiological mechanisms at play and attempts to identify the site of major resistance to aqueous outflow, Table 3. This classification scheme helps direct therapy against the inciting factors such as inflammation or neovascularization. Identifying the site of major resistance may also help select the optimal therapy for patients. For example, cases of glaucoma where most of the resistance is felt to be at the level of the trabecular meshwork may benefit from an SCD. However, in cases of raised EVP, the use of SCDs may theoretically predispose the patient toward hyphema postoperatively; therefore cases with raised EVP should not be managed with a SCD.

*Takeaway Points*

- PGAs are reasonable first-line therapies for a majority of patients. Initiation with combination therapy may be appropriate in select patients.
- Fixed-dose combinations are preferred second-line therapy over the addition of a single therapy.
  - Selection of fixed-dose combination should be based on required IOP reduction, 24-hour IOP profile, adverse effects, intolerance, concomitant disease, cost, or patient preference.
- SLT could be used earlier rather than later in the treatment algorithm.
- MIGS may be used to avoid a more invasive surgical intervention in patients with early to moderate stages of the disease, to improve patients' adherence to treatment, and quality of life.
- Patient and disease characteristics should be considered at all stages of the treatment algorithm and the therapy should be individualized according to patient needs. In addition, at all stages of the treatment algorithm it is imperative to monitor for adverse effects as well as disease progression (RNFL/VF/disc).
- The monocular trial is not considered useful and we suggest performance of binocular trials. To obtain the most valid results, multiple pressure checks should be performed before and after starting a new therapy.

## 8. Ensuring Patient Adherence

Multiple lines of evidence demonstrate that most glaucoma patients do not take their medications as intended. Reported adherence to glaucoma medications varies between 5% and 80%, with data collected indirectly by questionnaires or directly with electronic monitoring devices [158]. According to Tsai et al. [159], barriers to adherence in glaucoma are complex and can be categorized as those related to situation or environment, patient lifestyle and beliefs, provider attitudes, and the medication itself.

The adverse effects of the therapy, particularly the discomfort caused by the ophthalmic preparations and frequency of their administration, are major contributors to nonadherence to prescribed therapy. As glaucoma is an asymptomatic, slowly progressive chronic condition, patients will probably not notice the benefits of therapy, especially in the early stages of the disease, and the inconvenience of taking the medications may seem worse than the disease itself [160]. Approximately 50% of those who start therapy on IOP-lowering medications discontinue them within six months [161]. Despite this figure, many patients with glaucoma, especially those who were newly prescribed glaucoma medications, overestimate their medication adherence [162].

Because nonadherence to IOP-lowering medications puts patients at risk of not only disease progression but also to being prescribed additional glaucoma medications, regular and accurate assessment of medication adherence in clinical practice is essential. Various methods have been used to assess the medication adherence of patients with glaucoma including pharmacy refill methods [160], electronic monitoring [163, 164], and self-reporting measures [165, 166]. Estimates of adherence vary based on the assessment method, with pharmacy refill records producing the lowest and patient self-reporting the highest estimates of glaucoma medication adherence [166]. Several studies examining the validity of self-reported measures against objective measures (e.g., pharmacy records or electronic monitors) in patients with glaucoma supported the point that patients tend to overestimate their adherence to glaucoma medications [164, 165].

Although numerous studies have investigated factors that negatively affect patient compliance, physicians remain unable to significantly predict which patients will be adherent [167]. According to a recent Canadian study, fewer medications, use of prostaglandin analogues or *β*-blockers, living alone, and being widowed were associated with improved adherence [167]. Loon et al. [168] found that reduced belief in the need for treatment and higher degree of concern about adverse effects of the therapy are key factors associated with nonadherence. On the other hand, patients who have a stronger belief in the necessity for eye drops are more adherent [169]. Techniques targeting patient beliefs have been effective in improving adherence [170, 171].

Several studies have been conducted to assess measures that can be used to improve patient compliance. While a beneficial effect of patient education has often been presumed [158], it has been demonstrated that education alone is insufficient in improving adherence [166, 172]. Patient adherence is positively influenced by programs that couple education with other interventions (i.e., strategies for incorporating medication administration into daily activities) [173–176]. A recent review showed that interventions involving simplified dosing regimens, reminder devices, education, and individualized care planning improve adherence rates [177]. As revealed by Sleath et al. [178], provider communication behaviours, including the provision of education and positive reinforcement, can improve patient adherence to glaucoma medications. Individualized assessment is the first step in provider-patient communication and involves exploring the patient's personal and cultural perspective and beliefs. This might be of particular importance as some patients might opt for alternative therapies and neglect those prescribed by the ophthalmologist [179, 180]. In this case, the ophthalmologist needs to be prepared to explain to the patient the evidence behind approved and alternative therapies. He or she must motivate patients to take their prescribed medications and give them positive reinforcement.

Although one might expect advances in technology and communication to be useful in enhancing compliance, a recent study found that e-mail and text messaging reminders may have a limited utility in improving adherence in the general glaucoma population but may be useful for younger patients with glaucoma [181].

Timing of administration (i.e., morning or evening) can also make a difference for some patients. According to Ford et al. [182], patients prescribed a PGA for glaucoma prefer morning administration to evening administration. Inability to correctly administer the eye drops is another frequently observed problem in patients with glaucoma, including difficulty in aiming the drop, squeezing the bottle, and seeing the tip of the bottle. Thus, patients often rely on partners or relatives to administer the drops for them [183, 184]. Correct instillation of eye drops by glaucoma patients themselves, thus eliminating dependence on others, could be useful in improving adherence. In addition, it is probably beneficial to consider fixed combination therapies in patients requiring more than one type of medication.

*Takeaway Points*

- Poor adherence to treatment in glaucoma is believed to be one of the major reasons for treatment failure.
- Physician-patient communication appears to be a crucial factor in ensuring proper adherence to prescribed therapy.
- Interventions involving simplified dosing regimens, reminder devices, education, and individualized care planning can improve adherence rates.

## 9. Conclusions

Management of glaucoma in Canadian daily practice is undergoing significant changes in both diagnostic and treatment perspectives, with novel techniques complementing traditional approaches. This shift is resulting in earlier and more precise diagnosis that can lead to more effective treatments. When selecting the appropriate therapeutic targets, it is imperative to keep in mind individual patient characteristics and adapt the treatment according to the needs and preferences of patients and their care partners. This is of particular importance in patients with evidence of progressive disease where more aggressive therapeutic approaches and frequent therapy adjustment are required until the targeted (usually lower) IOP range is reached. Frequent assessment and follow-up and ongoing physician-patient dialogue are key to ensuring that the patient remains adherent to the prescribed therapy and that therapeutic goals are met.

## Figures and Tables

**Table:** (a) Open-angle glaucoma reproduced from [1], with permission from Elsevier

Suspect	One or two of the following: (1) IOP > 21 mm Hg (2) Suspicious disc or C/D asymmetry of >0.2 (3) Suspicious 24-2 (or similar) VF defect

Early	(1) Early glaucomatous disc features (e.g., C/D^*∗*^ < 0.65) (2) Mild VF defect not within 10° of fixation (e.g., MD better than −6 dB on HVF 24-2)

Moderate	(1) Moderate glaucomatous disc features (e.g., vertical C/D^*∗*^ 0.7–0.85) (2) Moderate VF defect not within 10° of fixation (e.g., MD from −6 to −12 dB on HVF 24-2)

Severe	(1) Advanced glaucomatous disc features (e.g., C/D^*∗*^ > 0.9) (2) VF defect within 10° of fixation^†^ (e.g., MD worse than −12 dB on HVF 24-2)

C/D, cup-to-disc; HVF, Humphrey visual field; IOP, intraocular pressure; MD, mean deviation; VF, visual field.

*∗* refers to vertical C/D ratio in an average size nerve. If the nerve is small, then a smaller C/D ratio may still be significant; conversely, a large nerve may have a large vertical C/D ratio and still be within normal limits. † also considers baseline 10-2 VF (or similar).

*Recent evidence indicates that ganglion cell loss should also be taken into consideration when staging/classifying glaucoma* [2].

**Table:** (b) Angle closure

Disease staging	≥180° appositionally closed (where posterior TM not visible [grade 1])	Ocular hypertension and/or peripheral anterior synechiae	Ganglion cell, optic nerve, and visual field damage
PACS	+	−	−
PAC	+	+	−
PACG	+	+	+

PAC, primary angle closure; PACS, primary angle closure suspect; PACG, primary angle closure glaucoma.

The above definitions could be used to guide decisions regarding the target IOP range (i.e., for patients with more advanced disease and/or evidence of rapidly progressing disease, targeted IOP should be in the lower targeted range). To achieve this, patients may require a more aggressive therapy from initiation of therapy; see Figure 3, suggested range for initial target IOP, and Figure 5, proposed treatment algorithm.

**Table 2:** Differences in IOP between different tonometers and Goldmann applanation tonometry [3].

Tonometer	Mean difference between tonometer and GAT	95% confidence interval		95% limits of agreement		% within 2 mmHg
DCT	1.8	+1.3	+2.3	−3.0	+6.6	47
NCT	0.3	−0.1	+0.7	−3.5	+4.0	69
ORA	1.5	+0.9	+2.2	−4.3	+7.3	45
Ocuton S	2.7	−1.2	+6.7	−4.0	+9.6	33
RT-(Icare)	0.9	+0.5	+1.5	−4.3	+6.3	51
TonoPen	0.2	−0.4	+0.9	−5.2	+5.7	52
Transpalpebral	−0.5	−1.3	+0.3	−7.0	+5.9	45

DCT, dynamic contour tonometer; NCT, noncontact tonometer; ORA, ocular response analyzer; RT, rebound tonometer.

Reproduced from [3] with permission from the European Glaucoma Society.

**Table 3:** Revised classification of glaucoma subtypes.

Primary/secondary classification	Angle status: open/angle closure	Etiology	Site of most resistance
Primary	Open	Ocular hypertensive	TM
Primary	Open	Ocular normotensive	TM
Secondary	Open	Inflammatory	TM/PostTM
Secondary	Open	Neovascular	TM/PostTM
Secondary	Open	TM obstructive (PXF, PDG, hemolytic, melanomalytic, phacolytic)	TM
Secondary	Open	Steroid-induced	TM
Secondary	Open	Raised PostTM outflow resistance	PostTM
Primary	Angle closure	Phacomorphic, plateau, pupillary block	PreTM
Secondary	Angle closure	Anterior pulling (ICE, PPMD, NVG, fibrous ingrowth, inflammatory)	PreTM
Secondary	Angle closure	Posterior pushing (tumor, choroidal effusion, ciliary body block)	PreTM

TM, trabecular meshwork; IES, iridocorneal endothelial syndrome; NVG, neovascular glaucoma; PPMD, posterior polymorphous dystrophy; PXF, pseudoexfoliation; PDG, pigment dispersion glaucoma.

## References

[1] (2009). Canadian Ophthalmological Society evidence-based clinical practice guidelines for the management of glaucoma in the adult eye. *Canadian Journal of Ophthalmology*.

[2] Medeiros F. A., Lisboa R., Weinreb R. N., Liebmann J. M., Girkin C., Zangwill L. M. (2013). Retinal ganglion cell count estimates associated with early development of visual field defects in glaucoma. *Ophthalmology*.

[3] European Glaucoma Society (2015). *Terminology and Guidelines for Glaucoma*.

[4] Gupta N., Yücel Y. H. (2007). Glaucoma as a neurodegenerative disease. *Current Opinion in Ophthalmology*.

[5] Na J. H., Lee K., Lee J. R., Baek S., Yoo S. J., Kook M. S. (2013). Detection of macular ganglion cell loss in preperimetric glaucoma patients with localized retinal nerve fibre defects by spectral-domain optical coherence tomography. *Clinical and Experimental Ophthalmology*.

[6] Lisboa R., Leite M. T., Zangwill L. M., Tafreshi A., Weinreb R. N., Medeiros F. A. (2012). Diagnosing preperimetric glaucoma with spectral domain optical coherence tomography. *Ophthalmology*.

[7] Singh K., Greenfield D. S. PPG: to treat or not to treat. http://ophthalmologytimes.modernmedicine.com/news/ppg-treat-or-not-treat?page=full.

[8] Schlötzer-Schrehardt U., Pasutto F., Sommer P. (2008). Genotype-correlated expression of lysyl oxidase-like 1 in ocular tissues of patients with pseudoexfoliation syndrome/glaucoma and normal patients. *The American Journal of Pathology*.

[9] Gemenetzi M., Yang Y., Lotery A. J. (2012). Current concepts on primary open-angle glaucoma genetics: a contribution to disease pathophysiology and future treatment. *Eye*.

[10] Kumar A., Basavaraj M. G., Gupta S. K. (2007). Role of CYP1B1, MYOC, OPTN and OPTC genes in adult-onset primary open-angle glaucoma: predominance of CYP1B1 mutations in Indian patients. *Molecular Vision*.

[11] Mantravadi A. V., Vadhar N. (2015). Glaucoma. *Primary Care: Clinics in Office Practice*.

[12] Mi X.-S., Yuan T.-F., So K.-F. (2014). The current research status of normal tension glaucoma. *Clinical Interventions in Aging*.

[13] Mroczkowska S., Benavente-Perez A., Negi A., Sung V., Patel S. R., Gherghel D. (2013). Primary open-angle glaucoma vs normal-tension glaucoma: the vascular perspective. *JAMA Ophthalmology*.

[14] Mudumbai R. C. (2013). Clinical update on normal tension glaucoma. *Seminars in Ophthalmology*.

[15] Song B. J., Caprioli J. (2014). New directions in the treatment of normal tension glaucoma. *Indian Journal of Ophthalmology*.

[16] Ghanem A. A., Elewa A. M., Arafa L. F. (2011). Endothelin-1 and nitric oxide levels in patients with glaucoma. *Ophthalmic Research*.

[17] Galassi F., Giambene B., Varriale R. (2011). Systemic vascular dysregulation and retrobulbar hemodynamics in normal-tension glaucoma. *Investigative Ophthalmology & Visual Science*.

[18] Grus F. H., Joachim S. C., Hoffmann E. M., Pfeiffer N. (2004). Complex autoantibody repertoires in patients with glaucoma. *Molecular Vision*.

[19] Wax M. B., Barrett D. A., Pestronk A. (1994). Increased incidence of paraproteinemia and autoantibodies in patients with normal-pressure glaucoma. *American Journal of Ophthalmology*.

[20] Berdahl J. P., Fautsch M. P., Stinnett S. S., Allingham R. R. (2008). Intracranial pressure in primary open angle glaucoma, normal tension glaucoma, and ocular hypertension: a case-control study. *Investigative Ophthalmology & Visual Science*.

[21] Mroczkowska S., Ekart A., Sung V. (2012). Coexistence of macro- and micro-vascular abnormalities in newly diagnosed normal tension glaucoma patients. *Acta Ophthalmologica*.

[22] Sergi M., Salerno D. E., Rizzi M. (2007). Prevalence of normal tension glaucoma in obstructive sleep apnea syndrome patients. *Journal of Glaucoma*.

[23] Wright C., Tawfik M. A., Waisbourd M., Katz L. J. (2016). Primary angle-closure glaucoma: an update. *Acta Ophthalmologica*.

[24] Quigley H. A., Broman A. T. (2006). The number of people with glaucoma worldwide in 2010 and 2020. *British Journal of Ophthalmology*.

[25] Quigley H. A., Friedman D. S., Congdon N. G. (2003). Possible mechanisms of primary angle-closure and malignant glaucoma. *Journal of Glaucoma*.

[26] Filho A. D., Cronemberger S., Mérula R. V., Calixto N. (2008). Plateau iris. *Arquivos Brasileiros de Oftalmologia*.

[27] Tarongoy P., Ho C. L., Walton D. S. (2009). Angle-closure glaucoma: the role of the lens in the pathogenesis, prevention, and treatment. *Survey of Ophthalmology*.

[28] Pavlin C. J., Harasiewicz K., Sherar M. D., Foster F. S. (1991). Clinical use of ultrasound biomicroscopy. *Ophthalmology*.

[29] Tham Y.-C., Li X., Wong T. Y., Quigley H. A., Aung T., Cheng C.-Y. (2014). Global prevalence of glaucoma and projections of glaucoma burden through 2040: a systematic review and meta-analysis. *Ophthalmology*.

[30] Cook C., Foster P. (2012). Epidemiology of glaucoma: What's new?. *Canadian Journal of Ophthalmology*.

[31] Buys Y. M. (2011). The need for ‘made in Canada’ glaucoma epidemiology data. *Canadian Journal of Ophthalmology*.

[32] Anraku A., Jin Y.-P., Butty Z. (2011). The Toronto epidemiology glaucoma survey: a pilot study. *Canadian Journal of Ophthalmology*.

[33] Perruccio A. V., Badley E. M., Trope G. E. (2007). Self-reported glaucoma in Canada: findings from population-based surveys, 1994–2003. *Canadian Journal of Ophthalmology*.

[34] Buys Y. M., Gaspo R., Kwok K. (2012). Referral source, symptoms, and severity at diagnosis of ocular hypertension or open-angle glaucoma in various practices. *Canadian Journal of Ophthalmology*.

[35] Buys Y. M., Jin Y.-P. (2013). Socioeconomic status as a risk factor for late presentation of glaucoma in Canada. *Canadian Journal of Ophthalmology*.

[36] Chen P. P. (2003). Blindness in patients with treated open-angle glaucoma. *Ophthalmology*.

[37] Lee P. P., Walt J. G., Doyle J. J. (2006). A multicenter, retrospective pilot study of resource use and costs associated with severity of disease in glaucoma. *Archives of Ophthalmology*.

[38] Einarson T. R., Vicente C., Machado M., Covert D., Trope G. E., Iskedjian M. (2006). Screening for glaucoma in Canada: a systematic review of the literature. *Canadian Journal of Ophthalmology*.

[39] Burr J. M., Mowatt G., Hernández R. (2007). The clinical effectiveness and cost-effectiveness of screening for open angle glaucoma: a systematic review and economic evaluation. *Health Technology Assessment*.

[40] Kassam F., Yogesan K., Sogbesan E., Pasquale L. R., Damji K. F. (2013). Teleglaucoma: improving access and efficiency for glaucoma care. *Middle East African Journal of Ophthalmology*.

[41] Hollands H., Johnson D., Hollands S., Simel D. L., Jinapriya D., Sharma S. (2013). Do findings on routine examination identify patients at risk for primary open-angle glaucoma? The rational clinical examination systematic review. *Journal of the American Medical Association*.

[42] Cross J. M., Girkin C. A., Owsley C., McGwin G. (2008). The association between thyroid problems and glaucoma. *British Journal of Ophthalmology*.

[43] Varma R., Lee P. P., Goldberg I., Kotak S. (2011). An assessment of the health and economic burdens of glaucoma. *American Journal of Ophthalmology*.

[44] Haymes S. A., Leblanc R. P., Nicolela M. T., Chiasson L. A., Chauhan B. C. (2007). Risk of falls and motor vehicle collisions in glaucoma. *Investigative Ophthalmology and Visual Science*.

[45] Skalicky S., Goldberg I. (2008). Depression and quality of life in patients with glaucoma: a cross-sectional analysis using the geriatric depression scale-15, assessment of function related to vision, and the glaucoma quality of life-15. *Journal of Glaucoma*.

[46] McKean-Cowdin R., Varma R., Wu J., Hays R. D., Azen S. P. (2007). Severity of visual field loss and health-related quality of life. *American Journal of Ophthalmology*.

[47] Rahman M. Q., Beard S. M., Discombe R., Sharma R., Montgomery D. M. I. (2013). Direct healthcare costs of glaucoma treatment. *British Journal of Ophthalmology*.

[48] Traverso C. E., Walt J. G., Kelly S. P. (2005). Direct costs of glaucoma and severity of the disease: a multinational long term study of resource utilisation in Europe. *British Journal of Ophthalmology*.

[49] Rein D. B., Zhang P., Wirth K. E. (2006). The economic burden of major adult visual disorders in the United States. *Archives of Ophthalmology*.

[50] Kitazawa Y., Horie T. (1975). Diurnal variation of intraocular pressure in primary open-angle glaucoma. *American Journal of Ophthalmology*.

[51] Newell F. W., Krill A. E. (1964). Diurnal tonography in normal and glaucomatous eyes. *Transactions of the American Ophthalmological Society*.

[52] Realini T., Weinreb N., Wisniewski S. (2011). Short-term repeatability of diurnal intraocular pressure patterns in glaucomatous individuals. *Ophthalmology*.

[53] Lee Y. R., Kook M. S., Joe S. G. (2012). Circadian (24-hour) pattern of intraocular pressure and visual field damage in eyes with normal-tension glaucoma. *Investigative Ophthalmology & Visual Science*.

[54] Renard E., Palombi K., Gronfier C. (2010). Twenty-four hour (Nyctohemeral) rhythm of intraocular pressure and ocular perfusion pressure in normal-tension glaucoma. *Investigative Ophthalmology and Visual Science*.

[55] Kent K. http://www.reviewofophthalmology.com/content/i/1533/c/28662.

[56] Susanna R., De Moraes C. G., Cioffi G. A., Ritch R. (2015). Why do people (still) go blind from glaucoma?. *Translational Vision Science and Technology*.

[57] Hatanaka M., Babic M., Susanna R. (2013). Reproducibility of the mean, fluctuation, and IOP peak in the diurnal tension curve. *Journal of Glaucoma*.

[58] Tajunisah I., Reddy S. C., Fathilah J. (2007). Diurnal variation of intraocular pressure in suspected glaucoma patients and their outcome. *Graefe's Archive for Clinical and Experimental Ophthalmology*.

[59] Kletke S. N., Varma D. K., Rai A. S., Ahmed I. K. (2014). *Proportion of Undetected Narrow Angles or Angle Closure in Cataract Surgery Referrals*.

[60] Baskaran M., Iyer J. V., Narayanaswamy A. K. (2015). Anterior segment imaging predicts incident gonioscopic angle closure. *Ophthalmology*.

[61] Vizzeri G., Weinreb R. N., Martinez de la Casa J. M. (2009). Clinicians agreement in establishing glaucomatous progression using the Heidelberg retina tomograph. *Ophthalmology*.

[62] Larocca F., Dhalla A.-H., Kelly M. P., Farsiu S., Izatt J. A. (2013). Optimization of confocal scanning laser ophthalmoscope design. *Journal of Biomedical Optics*.

[63] Alexandrescu C., Dascalu A. M., Panca A. (2010). Confocal scanning laser ophthalmoscopy in glaucoma diagnosis and management. *Journal of Medicine and Life*.

[64] Bussel I. I., Wollstein G., Schuman J. S. (2014). OCT for glaucoma diagnosis, screening and detection of glaucoma progression. *British Journal of Ophthalmology*.

[65] Le P. V., Tan O., Chopra V. (2013). Regional correlation among ganglion cell complex, nerve fiber layer, and visual field loss in glaucoma. *Investigative Ophthalmology and Visual Science*.

[66] Wessel J. M., Horn F. K., Tornow R. P. (2013). Longitudinal analysis of progression in glaucoma using spectral-domain optical coherence tomography. *Investigative Ophthalmology & Visual Science*.

[67] Medeiros F. A., Zangwill L. M., Bowd C., Mansouri K., Weinreb R. N. (2012). The structure and function relationship in glaucoma: implications for detection of progression and measurement of rates of change. *Investigative Ophthalmology and Visual Science*.

[68] Sung K. R., Sun J. H., Na J. H., Lee J. Y., Lee Y. (2012). Progression detection capability of macular thickness in advanced glaucomatous eyes. *Ophthalmology*.

[69] Vianna J. R., Danthurebandara V. M., Sharpe G. P. (2015). Importance of normal aging in estimating the rate of glaucomatous neuroretinal rim and retinal nerve fiber layer loss. *Ophthalmology*.

[70] Delgado M. F., Nguyen N. T. A., Cox T. A. (2002). Automated perimetry: a report by the american academy of ophthalmology. *Ophthalmology*.

[71] Medeiros F. A., Sample P. A., Weinreb R. N. (2004). Frequency doubling technology perimetry abnormalities as predictors of glaucomatous visual field loss. *American Journal of Ophthalmology*.

[72] Landers J. A., Goldberg I., Graham S. L. (2003). Detection of early visual field loss in glaucoma using frequency-doubling perimetry and short-wavelength automated perimetry. *Archives of Ophthalmology*.

[73] Traynis I., De Moraes C. G., Raza A. S., Liebmann J. M., Ritch R., Hood D. C. (2014). Prevalence and nature of early glaucomatous defects in the central 10° of the visual field. *JAMA Ophthalmology*.

[74] Chauhan B. C., Garway-Heath D. F., Goñi F. J. (2008). Practical recommendations for measuring rates of visual field change in glaucoma. *British Journal of Ophthalmology*.

[75] Quaranta L., Gandolfo F., Turano R. (2006). Effects of topical hypotensive drugs on circadian IOP, blood pressure, and calculated diastolic ocular perfusion pressure in patients with glaucoma. *Investigative Ophthalmology and Visual Science*.

[76] Ren R., Jonas J. B., Tian G. (2010). Cerebrospinal fluid pressure in glaucoma: a prospective study. *Ophthalmology*.

[77] Caprioli J., de Leon J. M., Azarbod P. (2016). Trabeculectomy can improve long-term visual function in glaucoma. *Ophthalmology*.

[78] American Academy of Ophthalmology Glaucoma Panel Preferred Practice Pattern® Guidelines. http://www.aao.org/ppp.

[79] Marvasti A. H., Tatham A. J., Zangwill L. M. (2013). The relationship between visual field index and estimated number of retinal ganglion cells in glaucoma. *PLoS ONE*.

[80] Meira-Freitas D., Lisboa R., Tatham A. (2013). Predicting progression in glaucoma suspects with longitudinal estimates of retinal ganglion cell counts. *Investigative Ophthalmology & Visual Science*.

[81] Leske M. C., Heijl A., Hussein M., Bengtsson B., Hyman L., Komaroff E. (2003). Factors for glaucoma progression and the effect of treatment: the Early Manifest Glaucoma Trial. *Archives of Ophthalmology*.

[82] Heijl A., Leske M. C., Bengtsson B., Hyman L., Bengtsson B., Hussein M. (2002). Reduction of intraocular pressure and glaucoma progression: results from the Early Manifest Glaucoma Trial. *Archives of Ophthalmology*.

[83] AGIS Investigators (2000). The Advanced Glaucoma Intervention Study (AGIS): 7. The relationship between control of intraocular pressure and visual field deterioration. *American Journal of Ophthalmology*.

[84] Vianna J. R., Chauhan B. C. (2015). How to detect progression in glaucoma. *Progress in Brain Research*.

[85] Chauhan B. C., Mikelberg F. S., Artes P. H. (2010). Canadian glaucoma study: 3. Impact of risk factors and intraocular pressure reduction on the rates of visual field change. *Archives of Ophthalmology*.

[86] Garway-Heath D. F., Crabb D. P., Bunce C. (2015). Latanoprost for open-angle glaucoma (UKGTS): a randomised, multicentre, placebo-controlled trial. *The Lancet*.

[87] Eyawo O., Nachega J., Lefebvre P. (2009). Efficacy and safety of prostaglandin analogues in patients with predominantly primary open-angle glaucoma or ocular hypertension: a meta-analysis. *Clinical Ophthalmology*.

[88] Quaranta L., Riva I., Katsanos A., Floriani I., Centofanti M., Konstas A. G. P. (2015). Safety and efficacy of travoprost solution for the treatment of elevated intraocular pressure. *Clinical Ophthalmology*.

[89] Riva I., Katsanos A., Floriani I. (2014). Long-term 24-hour intraocular pressure control with travoprost monotherapy in patients with primary open-angle glaucoma. *Journal of Glaucoma*.

[90] Alm A., Grierson I., Shields M. B. (2008). Side effects associated with prostaglandin analog therapy. *Survey of Ophthalmology*.

[91] Shah M., Lee G., Lefebvre D. R. (2013). A cross-sectional survey of the association between bilateral topical prostaglandin analogue use and ocular adnexal features. *PLoS ONE*.

[92] Khouri A., Lama P. J., Fechtner R. D., Netland P. A. (2008). Beta blockers. *Glaucoma Medical Therapy: Principles and Management*.

[93] Lama P. J. (2002). Systemic adverse effects of beta-adrenergic blockers: an evidence-based assessment. *American Journal of Ophthalmology*.

[94] Salpeter S., Ormiston T., Salpeter E. (2005). Cardioselective beta-blockers for chronic obstructive pulmonary disease. *Cochrane Database of Systematic Reviews*.

[95] Neufeld A. H., Zawistowski K. A., Page E. D., Bromberg B. B. (1978). Influences on the density of *β*-adrenergic receptors in the cornea and iris-ciliary body of the rabbit. *Investigative Ophthalmology and Visual Science*.

[96] Samama P., Bond R. A., Rockman H. A., Milano C. A., Lefkowitz R. J. (1997). Ligand-induced overexpression of a constitutively active *β*
_2_-adrenergic receptor: pharmacological creation of a phenotype in transgenic mice. *Proceedings of the National Academy of Sciences of the United States of America*.

[97] Molinoff P. B., Aarons R. D. (1983). Effects of drugs on *β*-adrenergic receptors on human lymphocytes. *Journal of Cardiovascular Pharmacology*.

[98] Maclure G. M. (1983). Chronic open angle glaucoma treated with timolol. A four year study. *Transactions of the Ophthalmological Societies of the United Kingdom*.

[99] Schuman J. S., Horwitz B., Choplin N. T., David R., Albracht D., Chen K. (1997). A 1-year study of brimonidine twice daily in glaucoma and ocular hypertension. A controlled, randomized, multicenter clinical trial. *Archives of Ophthalmology*.

[100] Ericson L. A. (1958). Twenty-four hourly variations of the aqueous flow. Examinations with perilimbal suction cup. *Acta Ophthalmologica*.

[101] Drance S. M. (1960). The significance of the diurnal tension variations in normal and glaucomatous eyes. *Archives of Ophthalmology*.

[102] Letchinger S. L., Frohlichstein D., Glieser D. K. (1993). Can the concentration of timolol or the frequency of its administration be reduced?. *Ophthalmology*.

[103] Ong L. B., Liza-Sharmini A. T., Chieng L. L. (2005). The efficacy of timolol in gel-forming solution after morning or evening dosing in Asian glaucomatous patients. *Journal of Ocular Pharmacology and Therapeutics*.

[104] Fudemberg S. J., Batiste C., Katz L. J. (2008). Efficacy, safety, and current applications of brimonidine. *Expert Opinion on Drug Safety*.

[105] van der Valk R., Webers C. A. B., Schouten J. S. A. G., Zeegers M. P., Hendrikse F., Prins M. H. (2005). Intraocular pressure-lowering effects of all commonly used glaucoma drugs: a meta-analysis of randomized clinical trials. *Ophthalmology*.

[106] Strom B. L., Schinnar R., Apter A. J. (2003). Absence of cross-reactivity between sulfonamide antibiotics and sulfonamide nonantibiotics. *The New England Journal of Medicine*.

[107] Johnson K. K., Green D. L., Rife J. P., Limon L. (2005). Sulfonamide cross-reactivity: fact or fiction?. *Annals of Pharmacotherapy*.

[108] Brackett C. C., Singh H., Block J. H. (2004). Likelihood and mechanisms of cross-allergenicity between sulfonamide antibiotics and other drugs containing a sulfonamide functional group. *Pharmacotherapy*.

[109] National Institute for Health and Care Excellence https://www.nice.org.uk/guidance/cg85.

[110] Kass M. A., Heuer D. K., Higginbotham E. J. (2002). The Ocular Hypertension Treatment Study: a randomized trial determines that topical ocular hypotensive medication delays or prevents the onset of primary open-angle glaucoma. *Archives of Ophthalmology*.

[111] Quaranta L., Biagioli E., Riva I. (2013). Prostaglandin analogs and timolol-fixed versus unfixed combinations or monotherapy for open-angle glaucoma: a systematic review and meta-analysis. *Journal of Ocular Pharmacology and Therapeutics*.

[112] Chrai S. S., Makoid M. C., Eriksen S. P., Robinson J. R. (1974). Drop size and initial dosing frequency problems of topically applied ophthalmic drugs. *Journal of Pharmaceutical Sciences*.

[113] Holló G., Topouzis F., Fechtner R. D. (2014). Fixed-combination intraocular pressure-lowering therapy for glaucoma and ocular hypertension: advantages in clinical practice. *Expert Opinion on Pharmacotherapy*.

[114] Jaenen N., Baudouin C., Pouliquen P., Manni G., Figueiredo A., Zeyen T. (2007). Ocular symptoms and signs with preserved and preservative-free glaucoma medications. *European Journal of Ophthalmology*.

[115] Pisella P. J., Pouliquen P., Baudouin C. (2002). Prevalence of ocular symptoms and signs with preserved and preservative free glaucoma medication. *British Journal of Ophthalmology*.

[116] Boimer C., Birt C. M. (2013). Preservative exposure and surgical outcomes in glaucoma patients: the PESO study. *Journal of Glaucoma*.

[117] Krupin T., Liebmann J. M., Greenfield D. S., Ritch R., Gardiner S. (2011). A randomized trial of brimonidine versus timolol in preserving visual function: results from the low-pressure glaucoma treatment study. *American Journal of Ophthalmology*.

[118] Quaranta L., Katsanos A., Floriani I., Riva I., Russo A., Konstas A. G. P. (2012). Circadian intraocular pressure and blood pressure reduction with timolol 0.5% solution and timogel 0.1% in patients with primary open-angle glaucoma. *Journal of Clinical Pharmacology*.

[119] Quaranta L., Miglior S., Floriani I., Pizzolante T., Konstas A. G. P. (2008). Effects of the timolol-dorzolamide fixed combination and latanoprost on circadian diastolic ocular perfusion pressure in glaucoma. *Investigative Ophthalmology and Visual Science*.

[120] Konstas A. G. P., Quaranta L., Yan D. B. (2012). Twenty-four hour efficacy with the dorzolamide/timolol-fixed combination compared with the brimonidine/timolol-fixed combination in primary open-angle glaucoma. *Eye*.

[121] Bournias T. E., Lai J. (2009). Brimonidine tartrate 0.15%, dorzolamide hydrochloride 2%, and brinzolamide 1% compared as adjunctive therapy to prostaglandin analogs. *Ophthalmology*.

[122] Day D. G., Hollander D. A. (2008). Brimonidine purite 0.1% versus brinzolamide 1% as adjunctive therapy to latanoprost in patients with glaucoma or ocular hypertension. *Current Medical Research and Opinion*.

[123] Reis R., Queiroz C. F., Santos L. C., Avila M. P., Magacho L. (2006). A randomized, investigator-masked, 4-week study comparing timolol maleate 0.5%, brinzolamide 1%, and brimonidine tartrate 0.2% as adjunctive therapies to travoprost 0.004% in adults with primary open-angle glaucoma or ocular hypertension. *Clinical Therapeutics*.

[124] Nguyen Q. H., McMenemy M. G., Realini T., Whitson J. T., Goode S. M. (2013). Phase 3 randomized 3-month trial with an ongoing 3-month safety extension of fixed-combination brinzolamide 1%/brimonidine 0.2%. *Journal of Ocular Pharmacology and Therapeutics*.

[125] Katz G., DuBiner H., Samples J., Vold S., Sall K. (2013). Three-month randomized trial of fixed-combination brinzolamide, 1%, and brimonidine, 0.2%. *JAMA Ophthalmology*.

[126] Realini T., Nguyen Q. H., Katz G., Dubiner H. (2013). Fixed-combination brinzolamide 1%/brimonidine 0.2% vs monotherapy with brinzolamide or brimonidine in patients with open-angle glaucoma or ocular hypertension: results of a pooled analysis of two phase 3 studies. *Eye*.

[127] Aung T., Laganovska G., Hernandez Paredes T. J., Branch J. D., Tsorbatzoglou A., Goldberg I. (2014). Twice-daily brinzolamide/brimonidine fixed combination versus brinzolamide or brimonidine in open-angle glaucoma or ocular hypertension. *Ophthalmology*.

[128] Narayanaswamy A., Neog A., Baskaran M. (2007). A randomized, crossover, open label pilot study to evaluate the efficacy and safety of Xalatan® in comparison with generic Latanoprost (Latoprost) in subjects with primary open angle glaucoma or ocular hypertension. *Indian Journal of Ophthalmology*.

[129] Kahook M. Y., Fechtner R. D., Katz L. J., Noecker R. J., Ammar D. A. (2012). A comparison of active ingredients and preservatives between brand name and generic topical glaucoma medications using liquid chromatography-tandem mass spectrometry. *Current Eye Research*.

[130] Fiscella R. G., Gaynes B. I., Jensen M. (2001). Equivalence of generic and brand-name ophthalmic products. *American Journal of Health-System Pharmacy*.

[131] Mammo Z. N., Flanagan J. G., James D. F., Trope G. E. (2012). Generic versus brand-name North American topical glaucoma drops. *Canadian Journal of Ophthalmology*.

[132] Van Santvliet L., Ludwig A. (2004). Determinants of eye drop size. *Survey of Ophthalmology*.

[133] Loftsson T., Jansook P., Stefánsson E. (2012). Topical drug delivery to the eye: dorzolamide. *Acta Ophthalmologica*.

[134] Tanihara H., Inoue T., Yamamoto T., Kuwayama Y., Abe H., Araie M. (2013). Phase 2 randomized clinical study of a Rho kinase inhibitor, k-115, in primary open-angle glaucoma and ocular hypertension. *American Journal of Ophthalmology*.

[135] Tanihara H., Inoue T., Yamamoto T. (2015). Additive intraocular pressure-lowering effects of the rho kinase inhibitor ripasudil (K-115) combined with timolol or latanoprost: a report of 2 randomized clinical trials. *JAMA Ophthalmology*.

[136] Tanihara H., Inoue T., Yamamoto T. (2016). One-year clinical evaluation of 0.4% ripasudil (K-115) in patients with open-angle glaucoma and ocular hypertension. *Acta Ophthalmologica*.

[137] Zhong Y., Yang Z., Huang W.-C., Luo X. (2013). Adenosine, adenosine receptors and glaucoma: an updated overview. *Biochimica et Biophysica Acta (BBA)—General Subjects*.

[138] Agarwal R., Agarwal P. (2014). Newer targets for modulation of intraocular pressure: focus on adenosine receptor signaling pathways. *Expert Opinion on Therapeutic Targets*.

[139] Karl M. O., Peterson-Yantorno K., Civan M. M. (2007). Cell-specific differential modulation of human trabecular meshwork cells by selective adenosine receptor agonists. *Experimental Eye Research*.

[140] Rasmussen C. A., Kaufman P. L., Ritch R., Haque R., Brazzell R. K., Vittitow J. L. (2014). Latrunculin B reduces intraocular pressure in human ocular hypertension and primary open-angle glaucoma. *Translational Vision Science & Technology*.

[141] Lusthaus J. A., Goldberg I. (2016). Emerging drugs to treat glaucoma: targeting prostaglandin F and E receptors. *Expert Opinion on Emerging Drugs*.

[142] Doozandeh A., Yazdani S. (2016). Neuroprotection in glaucoma. *Journal of Ophthalmic and Vision Research*.

[143] Tressler C. S., Beatty R., Lemp M. A. (2011). Preservative use in topical glaucoma medications. *Ocular Surface*.

[144] Noecker R., Miller K. V. (2011). Benzalkonium chloride in glaucoma medications. *Ocular Surface*.

[145] Mathews P. M., Ramulu P. Y., Friedman D. S., Utine C. A., Akpek E. K. (2013). Evaluation of ocular surface disease in patients with glaucoma. *Ophthalmology*.

[146] Konstas A. G. P., Quaranta L., Realini T. (2012). Overview of the BAK-free travoprost/timolol BAK-free fixed combination. *Expert Opinion on Pharmacotherapy*.

[147] Feldman R. M., Tanna A. P., Gross R. L. (2007). Comparison of the ocular hypotensive efficacy of adjunctive brimonidine 0.15% or brinzolamide 1% in combination with travoprost 0.004%. *Ophthalmology*.

[148] O'Connor D. J., Martone J. F., Mead A. (2002). Additive intraocular pressure lowering effect of various medications with latanoprost. *American Journal of Ophthalmology*.

[149] Realini T. D. (2009). A prospective, randomized, investigator-masked evaluation of the monocular trial in ocular hypertension or open-angle glaucoma. *Ophthalmology*.

[150] Bhorade A. M., Wilson B. S., Gordon M. O. (2010). The utility of the monocular trial: data from the ocular hypertension treatment study. *Ophthalmology*.

[151] Krishna R., DeBry P. W., Waldman C. W., Koulen P. (2012). Comparing the efficacy of the monocular trial treatment paradigm with multiple measurements of intraocular pressure before and after treatment initiation in primary open-angle glaucoma. *Clinical Ophthalmology*.

[152] (1995). The Glaucoma Laser Trial (GLT) and glaucoma laser trial follow-up study: 7. Results. Glaucoma Laser Trial Research Group. *American Journal of Ophthalmology*.

[153] Katz L. J., Steinmann W. C., Kabir A., Molineaux J., Wizov S. S., Marcellino G. (2012). Selective laser trabeculoplasty versus medical therapy as initial treatment of glaucoma: a prospective, randomized trial. *Journal of Glaucoma*.

[154] Lee J. W. Y., Yau G. S. K., Yick D. W. F., Yuen C. Y. F. (2015). Micropulse laser trabeculoplasty for the treatment of open-angle glaucoma. *Medicine*.

[155] Brandão L. M., Grieshaber M. C. (2013). Update on minimally invasive glaucoma surgery (MIGS) and new implants. *Journal of Ophthalmology*.

[156] Roelofs K., Arora S., Dorey M. W. (2014). Implantation of 2 trabecular microbypass stents in a patient with primary open-angle glaucoma refractory to previous glaucoma-filtering surgeries. *Journal of Cataract and Refractive Surgery*.

[157] Grieshaber M. C., Stegmann R., Grieshaber H. R., Meyer P. (2015). Novel device for expanding Schlemm's canal: a morphological study. *British Journal of Ophthalmology*.

[158] Olthoff C. M. G., Schouten J. S. A. G., Van De Borne B. W., Webers C. A. B. (2005). Noncompliance with ocular hypotensive treatment in patients with glaucoma or ocular hypertension: an evidence-based review. *Ophthalmology*.

[159] Tsai J. C., McClure C. A., Ramos S. E., Schlundt D. G., Pichert J. W. (2003). Compliance barriers in glaucoma: a systematic classification. *Journal of Glaucoma*.

[160] Friedman D. S., Quigley H. A., Gelb L. (2007). Using pharmacy claims data to study adherence to glaucoma medications: methodology and findings of the Glaucoma Adherence and Persistency Study (GAPS). *Investigative Ophthalmology & Visual Science*.

[161] Schwartz G. F., Quigley H. A. (2008). Adherence and persistence with glaucoma therapy. *Survey of Ophthalmology*.

[162] Sayner R., Carpenter D. M., Blalock S. J. (2015). Accuracy of patient-reported adherence to glaucoma medications on a visual analog scale compared with electronic monitors. *Clinical Therapeutics*.

[163] Vrijens B., Urquhart J. (2014). Methods for measuring, enhancing, and accounting for medication adherence in clinical trials. *Clinical Pharmacology and Therapeutics*.

[164] Cook P. F., Schmiege S. J., Mansberger S. L., Kammer J., Fitzgerald T., Kahook M. Y. (2015). Predictors of adherence to glaucoma treatment in a multisite study. *Annals of Behavioral Medicine*.

[165] Cate H., Bhattacharya D., Clark A., Holland R., Broadway D. C. (2013). Patterns of adherence behaviour for patients with glaucoma. *Eye*.

[166] Domino F. J. (2005). Improving adherence to treatment for hypertension. *American Family Physician*.

[167] Djafari F., Lesk M. R., Harasymowycz P. J., Desjardins D., Lachaine J. (2009). Determinants of adherence to glaucoma medical therapy in a long-term patient population. *Journal of Glaucoma*.

[168] Loon S. C., Jin J., Jin Goh M. (2015). The relationship between quality of life and adherence to medication in glaucoma patients in Singapore. *Journal of Glaucoma*.

[169] Stryker J. E., Beck A. D., Primo S. A. (2010). An exploratory study of factors influencing glaucoma treatment adherence. *Journal of Glaucoma*.

[170] Kripalani S., Yao X., Haynes R. B. (2007). Interventions to enhance medication adherence in chronic medical conditions: a systematic review. *Archives of Internal Medicine*.

[171] Gray T. A., Fenerty C., Harper R. (2012). Individualised patient care as an adjunct to standard care for promoting adherence to ocular hypotensive therapy: an exploratory randomised controlled trial. *Eye*.

[172] Beckers H. J. M., Webers C. A. B., Busch M. J. W. M., Brink H. M. A., Colen T. P., Schouten J. S. A. G. (2013). Adherence improvement in Dutch glaucoma patients: a randomized controlled trial. *Acta Ophthalmologica*.

[173] Norell S. E. (1979). Improving medication compliance: a randomised clinical trial. *The British Medical Journal*.

[174] Okeke C. O., Quigley H. A., Jampel H. D. (2009). Interventions improve poor adherence with once daily glaucoma medications in electronically monitored patients. *Ophthalmology*.

[175] Sleath B., Blalock S. J., Carpenter D. M. (2015). Ophthalmologist-patient communication, self-efficacy, and glaucoma medication adherence. *Ophthalmology*.

[176] Dreer L. E., Owsley C., Campbell L., Gao L., Wood A., Girkin C. A. (2016). Feasibility, patient acceptability, and preliminary efficacy of a culturally informed, health promotion program to improve glaucoma medication adherence among African Americans: ‘Glaucoma Management Optimism for African Americans Living with Glaucoma’ (GOAL). *Current Eye Research*.

[177] Gray T. A., Orton L. C., Henson D., Harper R., Waterman H. (2009). Interventions for improving adherence to ocular hypotensive therapy. *Cochrane Database of Systematic Reviews*.

[178] Sleath B., Carpenter D. M., Blalock S. J. (2015). Applying the resources and supports in self-management framework to examine ophthalmologist-patient communication and glaucoma medication adherence. *Health Education Research*.

[179] Saeedi O. J., Luzuriaga C., Ellish N., Robin A. (2015). Potential limitations of e-mail and text messaging in improving adherence in glaucoma and ocular hypertension. *Journal of Glaucoma*.

[180] Glaucoma Research Foundation Alternative Therapies for Glaucoma. http://www.glaucoma.org/treatment/alternative-therapies-for-glaucoma.php.

[181] Rhee D. J., Spaeth G. L., Myers J. S. (2002). Prevalence of the use of complementary and alternative medicine for glaucoma. *Ophthalmology*.

[182] Ford B. A., Gooi M., Carlsson A., Crichton A. C. (2013). Morning dosing of once-daily glaucoma medication is more convenient and may lead to greater adherence than evening dosing. *Journal of Glaucoma*.

[183] Stein J. D., Shekhawat N., Talwar N., Balkrishnan R. (2015). Impact of the introduction of generic latanoprost on glaucoma medication adherence. *Ophthalmology*.

[184] Kholdebarin R., Campbell R. J., Jin Y.-P. (2008). Multicenter study of compliance and drop administration in glaucoma. *Canadian Journal of Ophthalmology*.

